# Estimating *Cryptosporidium* and *Giardia* disease burdens for children drinking untreated groundwater in a rural population in India

**DOI:** 10.1371/journal.pntd.0006231

**Published:** 2018-01-29

**Authors:** Miles E. Daniels, Woutrina A. Smith, Marion W. Jenkins

**Affiliations:** 1 Department of Veterinary Medicine and Epidemiology, School of Veterinary Medicine, University of California at Davis, Davis, California United States of America; 2 Department of Institute of Marine Sciences, University of California at Santa Cruz, Santa Cruz, California, United States of America, Affiliated with: Fisheries Ecology Division, Southwest Fisheries Science Center, National Marine Fisheries Service, National Oceanic and Atmospheric Administration, Santa Cruz, California, United States of America; 3 Department of Civil and Environmental Engineering, University of California at Davis, Davis, California, United States of America; Institut Pasteur, FRANCE

## Abstract

**Background:**

In many low-income settings, despite improvements in sanitation and hygiene, groundwater sources used for drinking may be contaminated with enteric pathogens such as *Cryptosporidium* and *Giardia*, which remain important causes of childhood morbidity. In this study, we examined the contribution of diarrhea caused by *Cryptosporidium* and *Giardia* found in groundwater sources used for drinking to the total burden of diarrheal disease among children < 5 in rural India.

**Methodology/Principal findings:**

We studied a population of 3,385 children < 5 years of age in 100 communities of Puri District, Odisha, India. We developed a coupled quantitative microbial risk assessment (QMRA) and susceptible-infected-recovered (SIR) population model based on observed levels of *Cryptosporidium* and *Giardia* in improved groundwater sources used for drinking and compared the QMRA-SIR estimates with independently measured all-cause (i.e., all fecal-oral enteric pathogens and exposure pathways) child diarrhea prevalence rates observed in the study population during two monsoon seasons (2012 and 2013). We used site specific and regional studies to inform assumptions about the human pathogenicity of the *Cryptosporidium* and *Giardia* species present in local groundwater. In all three human pathogenicity scenarios evaluated, the mean daily risk of *Cryptosporidium* or *Giardia* infection (0.06–1.53%), far exceeded the tolerable daily risk of infection from drinking water in the US (< 0.0001%). Depending on which protozoa species were present, median estimates of daily child diarrhea prevalence due to either *Cryptosporidium* or *Giardia* infection from drinking water was as high as 6.5% or as low as < 1% and accounted for at least 2.9% and as much as 65.8% of the all-cause diarrhea disease burden measured in children < 5 during the study period. *Cryptosporidium* tended to account for a greater share of estimated waterborne protozoa infections causing diarrhea than did *Giardia*. Diarrhea prevalence estimates for waterborne *Cryptosporidium* infection appeared to be most sensitive to assumptions about the probability of infection from ingesting a single parasite (i.e. the rate parameter in dose-response model), while *Giardia* infection was most sensitive to assumptions about the viability of parasites detected in groundwater samples.

**Conclusions/Significance:**

Protozoa in groundwater drinking sources in rural India, even at low concentrations, especially for *Cryptosporidium*, may account for a significant portion of child diarrhea morbidity in settings were tubewells are used for drinking water and should be more systematically monitored. Preventing diarrheal disease burdens in Puri District and similar settings will benefit from ensuring water is microbiologically safe for consumption and consistent and effective household water treatment is practiced.

## Author summary

Water, sanitation, and hygiene (WASH) interventions aimed at reducing exposure to enteric pathogens have produced mixed health impacts, with some interventions finding no significant difference in health outcomes between intervention and control groups. While there are many explanations why individual WASH interventions may not achieve improved health outcomes, one reason is an incomplete understanding of the conditions that favor perpetuation and transmission of enteric pathogens in a given population and region. In this study, we developed a set of diarrhea-causing disease transmission models using measurements of drinking water contamination and child diarrhea over the same time period in the same study population. Using the disease transmission models, we examined how much of the observed diarrhea in children was due to waterborne transmission of enteric pathogens in a program in rural India that improved household sanitation but failed to produce improvements in child health. We focused on the role of two enteric protozoal pathogens, *Cryptosporidium* and *Giardia*, and diarrhea rates among children < 5 years of age in these communities. We found that *Cryptosporidium* and *Giardia* infections from drinking water contaminated with these enteric protozoa may have together caused as much as 65.8% (IQR 63.4–68.2%) or as little as 2.9% (IQR 2.3–3.4%) of the observed diarrhea in children depending on modeling assumptions about which protozoa species were present. These findings suggest implementing a single barrier, such as only sanitation, to disrupt the multiple pathways of fecal-oral transmission of enteric pathogens, rather than multiple barriers, such as sanitation and safe drinking water, may lead some interventions to fall short of achieving measurable health improvements. Finally, our research suggests that *Cryptosporidium* and *Giardia* may cause significant amounts of child diarrhea morbidity even at low levels of concentration when present in improved drinking water sources and their measurement should be including in community drinking water quality monitoring programs.

## Introduction

Untreated groundwater is the primary source of drinking water for nearly a quarter of the world’s population and in low-income settings groundwater tubewells are widely used for drinking [1]. Although tubewells have been classified as an improved source of drinking water, they are susceptible to fecal contamination [2], making them a potential transmission pathway for diarrheal pathogens unless water is properly treated before ingestion. Globally, nearly 1.5 million deaths are estimated to occur each year from diarrheal diseases, with the majority of these deaths occurring in low-income settings and in children < 5 years old [3]. In low-income settings, however, fewer than half the population is reported to treat their drinking water at home to remove pathogens, and poorer households are the least likely to do so [4]. In rural India roughly 77% of people rely for drinking water on non-piped improved water sources (e.g. tubewells) [1], which raises concerns about exposure to waterborne pathogens caused by fecal contamination.

Etiology of childhood diarrhea is complex, but where surveillance occurs protozoal pathogens are recognized as important contributors to waterborne disease [5]. *Cryptosporidium* and *Giardia* are two fecal protozoal pathogens which cause diarrhea and both have zoonotic potential. The primary zoonotic species of *Cryptosporidium* and *Giardia* known to infect humans are *Cryptosporidium parvum* and *Giardia lamblia* (syn. *Giardia duodenalis* and *Giardia intestinalis*) assemblages A and B, respectively [6]. Additionally, *Cryptosporidium hominis* is a human-specific species [7]. These pathogens can be transmitted via contaminated drinking water as well as contaminated recreational or bathing water, food, soil, and hands, and are responsible for substantial disease burdens worldwide [8]. In developing countries *Cryptosporidium* and *Giardia* are frequently detected in stools of children in hospital- and community-based studies [9–11]. Additionally, *Cryptosporidium* is a leading cause of moderate to severe diarrhea in children < 2 years old in India [10] and both cryptosporidiosis and giardiasis have been associated with stunting, malnutrition, and wasting when diagnosed as a chronic disease [12–14]. When measured with methods able to detect relatively low concentrations, *Cryptosporidium* and *Giardia* have been frequently detected in water sources in India [15], pointing to potentially large but unknown risks.

Risk and simulation modeling are mathematical approaches used to estimate exposure risks and levels of disease burden attributed to environmental exposures, including infectious diseases. Quantitative microbial risk assessment (QMRA) is a method typically used to quantify risk of infection for target pathogens in drinking water and food [16]. Susceptible-infected-recovered (SIR) modeling is a population level simulation approach. In QMRA, the probability of an infection is modeled on an individual basis and determined by exposure conditions for a given exposure scenario, such as ingested volume of water and concentration of disease-causing pathogens in the ingested volume. With SIR models, an exposed individual is categorized as susceptible, infected, or recovered from an infection and their health status and exposure levels simulated over time. Rarely are data on both pathogen exposure and disease outcomes available in the same population at the same time to develop accurate models of exposure risks and compare model estimates to observed values. Most often modeling studies have information on disease burdens without pathogen exposure data [17, 18] or exposure data without levels of disease [19, 20]. This is a common weakness of many published modeling studies and results in an inability to compare modeling assumptions, risk results, or disease burden estimates to actual levels of disease.

The objective of this study was to better understand the public health significance of *Cryptosporidium* and *Giardia* contamination detected in groundwater sources used for drinking in a low-income population in rural Puri District, Odisha, India. To do so, we estimated the infection risk and associated child diarrhea disease burden from drinking groundwater and compared estimates to independently measured child diarrhea prevalence rates observed in the same population over the same period when contamination was observed. Specifically, we had three research questions: 1) what is the daily protozoal infection risk for children drinking from contaminated tubewells, 2) what is the associated child diarrhea disease burden in the study population, and 3) how does the estimated child diarrhea disease burden attributable to protozoa in drinking water compare to actual levels of all-pathogen/all-pathway child diarrhea observed in the study population, or stated alternatively, how much child diarrhea morbidity could be explained by consumption of protozoa-contaminated drinking water. To address these research questions, the prevalence of diarrhea in children < 5 years old from ingestion of *Cryptosporidium* and/or *Giardia* in contaminated tubewell drinking water was estimated using a QMRA approach coupled with a SIR population model and child diarrhea prevalence estimates compared to measured rates during the monsoon season in 2012 and 2013. The main inputs to the QMRA and SIR models were taken from previously published research in the study population as a part of a large sanitation intervention trial, including measured protozoal concentrations in tubewells [15], caretaker-reported 7-day recall diarrhea period prevalence [21], and site-specific population characteristics and data [22]. Sensitivity analysis was used to identify model inputs which were associated with the greatest uncertainty in the *Cryptosporidium* and *Giardia* attributable waterborne child diarrhea prevalence estimates.

## Methods

### Study site and population

Study villages were part of a large-scale cluster randomized control trial (the Odisha Sanitation Trial) evaluating the health impacts of a Total Sanitation Campaign program in 2011 [21]. Baseline data indicate the majority of households (82%) got their drinking water from tubewells, with 39% using deep public tubewells installed by local government and 43% using privately owned shallow tubewells installed by the private sector. Additional details of the study villages can be found elsewhere [22]. In the Sanitation Trial, a total of 3,835 children < 5 years old in 100 villages were enrolled in diarrhea surveillance monitoring in which caretakers reported 7-day recall period prevalence for child diarrhea measured once every three months on a rolling basis for a period of almost two years starting in 2012. Reported diarrhea episodes may have been caused by any number of pathogens circulating in the population since stool samples were not collected for pathogen screening.

### QMRA

**Fig 1:**
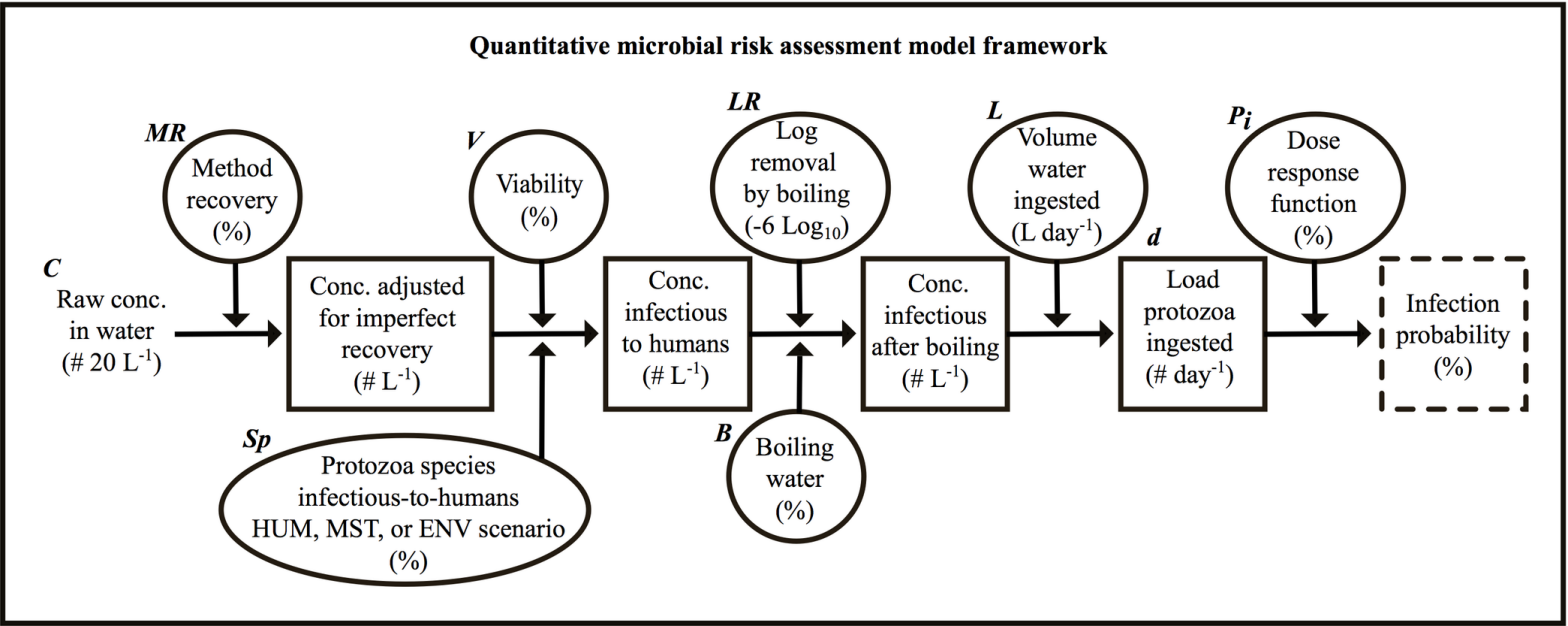
Conceptual framework of the quantitative microbial risk assessment model. Framework includes the computational process, parameters, and variables included in estimating risk of infection for *Cryptosporidium* or for *Giardia* from contaminated drinking water. The driving input parameter (raw concentration of parasites) is identified at the left most section of the figure. Boxes represent modeled variables (e.g., modified concentration and load of parasites), ovals represent exogenous parameters and assumptions affecting modeled variables (e.g. HUM, MST, and ENV scenarios represent the fraction of parasites belonging to a species infectious-to-humans), and the dashed box on the right represents the model output. Units are displayed within brackets under each description. Variable and parameter symbols shown directly above their respective variable or parameter are used in Eq 1, 2 and 4.

**Fig 2:**
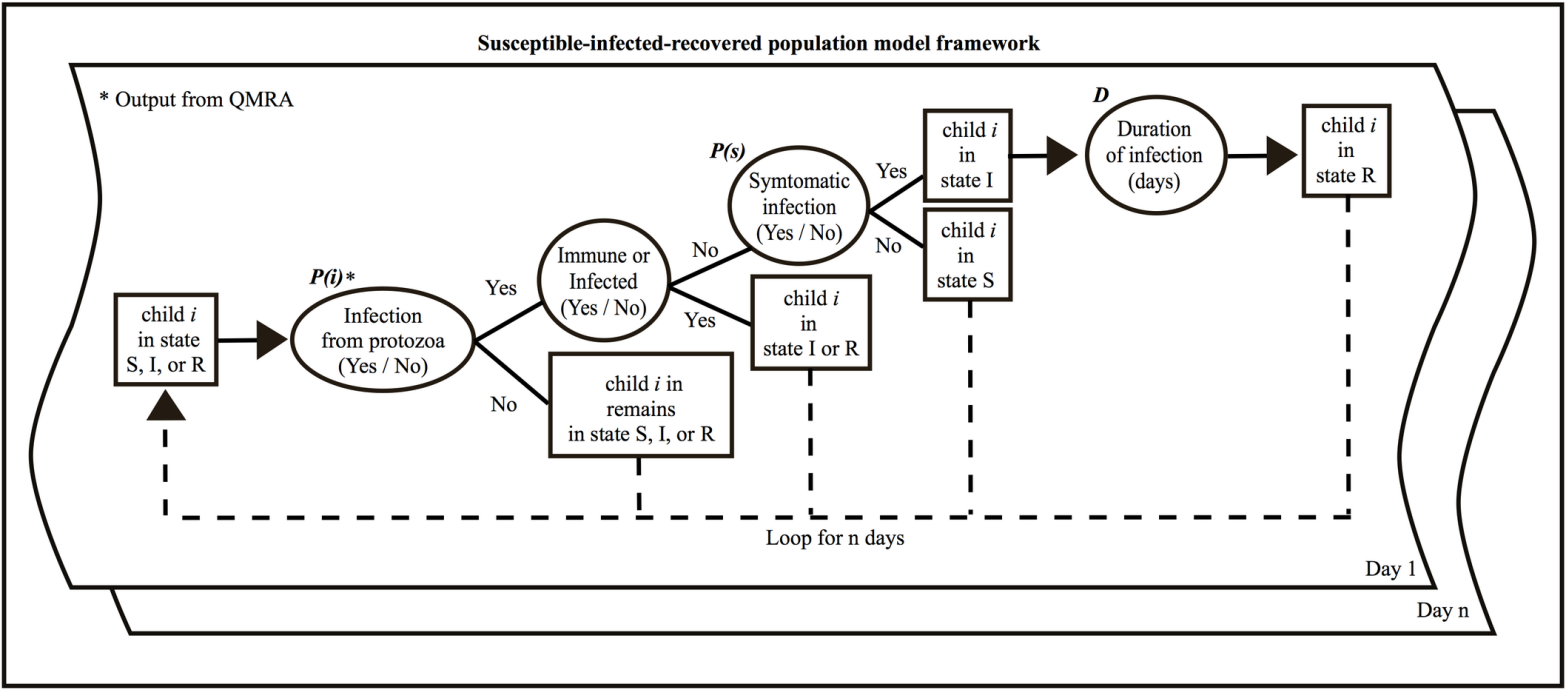
Conceptual framework of the susceptible-infected-recovered model. Framework includes the steps, parameters, and variables included in estimating diarrheic infections of *Cryptosporidium* and *Giardia* from contaminated drinking water for each individual (*i*) in the study population. Boxes represent modeled variables (e.g. state of child) and ovals represent exogenous parameters and assumptions affecting modeled variables, i.e. *P(i)*, *P(s)*, and *D*. Units are displayed within brackets under each description.

To estimate the probability of a symptomatic case of cryptosporidiosis or giardiasis from drinking water in children < 5 years old, and to estimate prevalence of diarrhea over time in the study population of 3,835 children in study communities exposed to contaminated tubewell drinking water, we used a quantitative microbial risk assessment (QMRA) approach [23] (Fig 1) coupled with a susceptible-infected-recovered (SIR) model (Fig 2). QMRA is composed of four main steps: (1) hazard identification, (2) dose-response, (3) exposure assessment, and (4) risk characterization. During hazard identification, information is gathered describing how a particular pathogen affects the population of interest. Typical information can include the population size and typical pathogen shedding rates from an infected host. During dose-response, a mathematical model is developed or selected to estimate the probability that an individual will become infected with a pathogen given a certain number of organisms ingested and characteristics of the pathogen. The goal of exposure assessment is to characterize the different processes that contribute to the number of organisms an individual is exposed to through an activity, such as volume of water intentionally ingested for hydration (i.e., daily drinking) purposes. Risk characterization is the stage where information from the three previous steps is synthesized to estimate infection and/or illness risk for an individual or a population.

In our QMRA, we used information about the hazard (i.e. contaminated drinking water), the exposure (i.e. amount drunk, amount treated, etc.), the target pathogen (i.e. *Cryptosporidium* and *Giardia*) and the host (i.e. children < 5), described by statistical distributions or point estimates, to estimate the probability that exposure resulted in ingestion of a target pathogen, and that the estimated amount ingested resulted in an infection for the host. To reflect the variability inherent in modeling disease transmission and infection from a drinking water source, given uncertain and variable concentrations of viable and infectious pathogens in the drinking water in space and time, we used Monte Carlo simulation to repeatedly and randomly sample the statistical distributions that characterize the amount of ingested target pathogen. All simulations were programmed and run using the R modeling environment [24]. The main parameters of the QMRA model, their values and distributions, and sources of data are summarized in Table 1. Each QMRA modeling step is described in detail next.

**Table 1:** Main parameters used in the quantitative microbial risk assessment and susceptible-infected-recovered models developed to simulate waterborne diarrheal infections in children < 5 in Puri District due to *Cryptosporidium* and *Giardia* in drinking water from deep (DTW) and shallow (STW) tubewells over two monsoon seasons.

Parameter Symbol (Fig 1): Description (distribution)	Parameter value(s) of distribution or point estimate	Source
**QMRA parameter set**		
***MR***: Method recovery (beta binomial) *Crypto*. mean = 55%, *Giardia* mean = 34%	*Crypto*. = α = 1.9, β = 1.9 *Giardia* = α = 3.0, β = 6.0	[15]
***V***: Viability (beta binomial) *Crypto*. mean = 38%, *Giardia* mean = 13%	*Crypto*. α = 1.65, β = 2.46 *Giardia* α = 2.93, β = 17.4	[25, 26]
***H (HUM scenario)***: Mean fraction of human parasites	*Crypto*. = 100%, *Giardia =* 100%	[15, 27–29]
***H (ENV scenario)***: Mean fraction of human parasites	*Crypto*. = 6%, *Giardia =* < 1%	[15, 27–29]
***H (MST scenario)***: Mean fraction of human parasites	DTW 2012 = 50%, STW 2012 = 16% DTW 2013 = 20%, STW 2013 = 33%	[15, 30–38]
$\hat{Z}$ ***(ENV and MST scenario)***: Mean fraction of non-human parasites infectious-to-humans [zoonotic faction]	*Crypto*. = 41%, *Giardia =* 19%	[15, 30–38]
***Sp (HUM*, *ENV*, *MST scenarios)***: Mean fraction of parasites of a species infectious-to-humans	$Sp=H+(1-H)\times \hat{Z}$	
***LR***: Reduction from boiling water	*Crypto*. = 6 Log_10_, *Giardia =* 6 Log_10_	[39]
***L***: Liters of water ingested per day (uniform)	a = 0.893, b = 1.39	[40, 41]
***r***: Exponential dose response parameter (triangular dist.)*	*Crypto*. = a = 0.00021, c = 0.0042, b = 0.0500 *Giardia =* a = 0.0097, c = 0.0198, b = 0.0358	[42–44]
**SIR parameter set**		
***TW***: Probability of using a particular tubewell type	DTW = *p* = 48% STW = *p* = 52%	Base line survey
***B***: Probability of boiling tubewell water before drinking (Bernoulli)	DTW = *p* = 9% STW = *p* = 16%	Base line survey
***P(s)***: Morbidity ratio for diarrheic infection (Bernoulli, where *p* is uniform**)	*Crypto*. = *p* = a = 29%, b = 68% *Giardia = p* = a = 49%, b = 59%	[9, 45–47]
***D***: Duration of illness (gamma) *Crypto*. mean = 10 days, *Giardia* mean = 11days	*Crypto*. = shape = 1.1, scale = 9.2 *Giardia* = shape = 3.2, scale = 3.4	[17, 48–51]
***Re***: Reduced recall of diarrhea 2 or more days after occurrence (Bernoulli, where *p* is uniform**)	*p* = a = 12%, b = 52%	[52]

* a, c, b for the triangular distribution are minimum, mode, and maximum respectively.

** a and b for uniform distribution are minimum and maximum.

#### Hazard identification

*Cryptosporidium* and *Giardia* are both fecal-oral pathogens with a variety of host species depending on the particular pathogen species, with oocysts and cysts representing the infectious stage of their life cycle respectively. Both of these protozoa have been detected in drinking water sources of the study population [15] and represent a potential hazard for consumers of untreated water. Some of the main symptoms in humans associated with *Cryptosporidium* and *Giardia* infections include diarrhea, bloating, cramps, and fatigue. However, depending on the immune status, prior exposure, and other factors, not all infected individuals will have the same probability of developing a diarrheic symptom (morbidity ratio). Additionally, not all cases of an infection will persist for the same period of time (illness duration). While interactions between the host and pathogen are complex, observational studies of children, especially those conducted in low-income settings, provide an estimate of the morbidity ratio, illness duration, and other characteristics of *Cryptosporidium* and *Giardia* infections relevant to our study population as presented in the SIR model section below.

#### Dose-response

The probability of infection after ingesting a given number of *Cryptosporidium* or *Giardia* parasites was determined using a dose-response (D-R) model [23]. Here, we assumed an exponential D-R model (Eq 1) with a dose variable, *d*, describing the number of parasites ingested and a rate parameter, *r*, describing the probability that each individual parasite in dose, *d*, results in an infection. For each protozoa, *r* was derived from published challenge studies in which volunteers were given a known dose of assumed viable *Cryptosporidium parvum* or *Giardia lamblia* and followed over time to determine if infection occurred [42–44]. We represented uncertainty in the D-R model related to differences in virulence between parasites, in susceptibility between hosts, and other factors, using upper and lower limits reported in challenge studies as bounds for a triangular distribution describing the rate parameter, *r*, and we set the mode to the best fit value reported in the challenge studies (Table 1). (see Appendix A in S1 Supporting Information for details).

$$P_{i}=1-e^{-rd}$$ (1)

where *P*_*i*_ is the probability of infection given the number of parasites ingested, *d*, and *r*, the rate parameter (see Fig 1). The closer *r* is to one, the greater the chance of infection for a given *d*.

#### Exposure assessment

Estimating daily exposure to *Cryptosporidium* and to *Giardia* (the daily dose, *d*) from drinking tubewell water involved three steps: 1) assigning a raw parasite concentration value (*C* in Fig 1), for tubewell water (see next section) and adjusting for method recovery efficiency (*MR*) and viability (*V*), 2) accounting for the species of pathogens infectious-to-humans (*Sp*), and 3) assigning a daily volume of tubewell water consumed for drinking (*L*) and accounting for removal of parasites by boiling (*B* and *LR*), to calculate an ingested dose (*d*) of viable parasites of a species infectious-to-humans. Each step is described in further detail below.

#### Protozoal concentrations in tubewells

As previously reported [15], *Cryptosporidium* and *Giardia* concentrations in deep and shallow tubewells were measured once in 206 tubewells in Odisha Sanitation Trial villages, during the monsoon season (June-August) of either 2012 or 2013. A 20 L sample of tubewell water was processed via ultrafiltration followed by immunomagnetic separation (IMS) and a direct immunofluorescent antibody (DFA) test to detect and enumerate *Cryptosporidium* and *Giardia*. IMS is specifically designed to target species of known public health concern, such as *C*. *hominis* and *Giardia lamblia*, but can also capture a variety of other species [53]. *Cryptosporidium* oocysts were detected in 14% of deep (n = 110) and 5% of shallow (n = 96) tubewells, while *Giardia* cysts were detected in 12% of deep (n = 110) and 17% shallow (n = 96) tubewells. Concentration data for each parasite, tubewell type, and year are shown in Table 2. A two-sample Kolmogorov-Smirnov test indicated concentrations were significantly different between years (*P-value* = < 0.001).

**Table 2:** Concentrations of *Cryptosporidium* oocysts and *Giardia* cysts detected in tubewells during the 2012 and 2013 monsoon season.

Water Source	Monsoon Year	Mean (range) *Cryptosporidium* oocysts 20 L^-1^	Mean (range) *Giardia* cysts 20 L^-1^
Deep tubewell	2012	11 (0–110)	20 (0–520)
	2013	< 1 (0–13)	< 1 (0–9)
Shallow tubewell	2012	5 (0–115)	12 (0–70)
	2013	2 (0–94)	4 (0–201)

We developed independent probability distributions for *Cryptosporidium* and *Giardia* concentrations by fitting a statistical distribution to the concentration data, which were non-negative, continuous, and typically skewed, using maximum likelihood methods. A gamma distribution of the protozoa concentration in tubewell water (# 20 L^-1^), with a shape and scale parameter, was chosen because it outperformed other models examined (i.e. Poisson) both in terms of capturing the spread of concentration data, as indicated by visual inspection, and maximum likelihood values. For model fitting purposes and to account for the lower limit of detection using IMS-DFA, tubewell samples below the sample limit of detection (i.e. non-detects) were given a value of ½ the sample limit of detection (i.e. 1 parasite per DFA slide well) (see Appendix B.1 in S1 Supporting Information). At most, the data replacement procedure resulted in non-detects being assigned a concentration of six parasites per 20 L of water (< 1 parasite per liter of water ingested) and likely had little impact on model results. To account for concentration differences between parasites (*Cryptosporidium* and *Giardia*), sample year (2012–2013), and tubewell type (deep *vs*. shallow), separate gamma distributions were fitted for each parasite, year, and tubewell type (see Appendix B.2 in S1 Supporting Information).

Spiking studies of *Cryptosporidium* and *Giardia* in water show method recovery efficiency can be highly variable [54]. This variability can have important consequences for QMRA when ignored and can result in a 100-times underestimation of concentration levels when method recovery is low. To characterize the method recovery of parasites in our study, we followed methods previously described [54], where a beta-binomial distribution was fit to data from spiking trials for each parasite using maximum likelihood methods (see Appendix B.3 in S1 Supporting Information). Briefly, spiking trials occurred 11 times over the course of collecting tubewell samples and consisted of adding 200 *Cryptosporidium* oocysts and 200 *Giardia* cysts to 20 L of DI water. Spiked samples were processed identically to tubewell samples. Each sampled value from the fitted protozoa concentration distribution in tubewells was adjusted by multiplying it by the reciprocal of a sampled value from the relevant fitted method recovery beta-binomial distribution (see Table 1). Mean method recovery was estimated to be 55% (IQR 33–72%) for *Cryptosporidium* and 34% (IQR 23–45%) for *Giardia*.

In addition to method recovery, the fraction of parasites able to result in a host infection (i.e. viable) when of a species infectious-to-humans can have important effects on estimates of disease and result in overestimation of risk when all parasites are assumed to be viable. Information on the viability of detected parasites in tubewell samples was not available for this study. To estimate the effect of viability, we used published studies that visually identified infectivity characteristics from 85 environmental water samples collected in North America [25, 26] as no published viability data was found for the study region. Similar to method recovery, viability was modeled as a beta-binomial distribution with an estimated mean viability of 38% (IQR 22–56%) and 13% (IQR 8–19%), respectively, for detected *Cryptosporidium* oocysts and *Giardia* cysts (see Table 1) as reported previously [25].

#### Fraction of detected parasites of a species infectious-to-humans

As tubewell samples may have contained a mixture of human and non-human shed parasites, we simulated three different scenarios to estimate the fraction of parasites detected in a tubewell sample that was a species infectious-to-humans (*Sp*), with *Sp* determined as follows:

$$Sp=H+(1-H)\times \hat{Z}$$ (2)

where *H* is the fraction of human shed parasites and $\hat{Z}$ is the fraction of non-human shed parasites of a species infectious-to-humans (i.e. zoonotic fraction), with *H* calculated for each year (2012–2013), tubewell type (deep and shallow), and parasite (*Cryptosporidium* and *Giardia*) when appropriate.

Scenario one (HUM) assumed humans shed all of the detected parasites and 100% of both parasites were species infectious-to-humans. Therefore, *H* = 1 always and $\hat{Z}$ is irrelevant in the calculation of *Sp*. The HUM scenario equated to the case with the highest level of parasites infectious-to-humans in tubewells and thus represented the scenario with the highest risk of infection.

Scenario two (ENV) assumed the probability of a parasite shed by a human host (*H*) was directly proportional to the ratio of the estimated environmental load of parasites shed by the population of humans to the total estimated environmental load shed by the population of humans, livestock, and domestic animals in Puri District [15]. Livestock included cattle, buffalo, sheep, and goats and domestic animals included dogs, as these represented the majority of animal species in the study region. To calculate the parasite environmental loading rates for humans and each animal species, we used four primary pieces of information as described previously [15]: parasite prevalence, parasite shedding rate, host population demographics, and host fecal production rates (see Appendix B.6.2 in S1 Supporting Information for further details).

Scenario three (MST) used microbial fecal source tracking (MST) results by year and tubewell type for the same protozoa-tested tubewell water samples [55]. MST uses molecular methods to detect enteric bacteria specific to a host to identify the likely source of fecal contamination. We assumed the probability of a parasite being shed by a human (*H*) was directly proportional to the ratio of the prevalence of human host-specific MST markers to the prevalence of non-human animal MST markers in tested tubewells (see *H* in Table 1 for MST scenario) (see Appendix B.6.3 in S1 Supporting Information for further details).

For both the ENV and MST scenarios, we assumed a portion of the non-human fraction was a species infectious-to-humans and we characterized this fraction using a point estimate termed the “zoonotic fraction” ($\hat{Z}$). The fraction of non-human parasites (shed by livestock and domestic animals) infectious-to-humans is affected by a number of factors relating to animal husbandry, cultural practices, host health status, and pathogen-host interactions, among others. To estimate the zoonotic fraction term, published literature on protozoa species found in fecal samples of each of the five-animal species of interest was used to estimate a mean prevalence of fecal samples containing zoonotic *C*. *parvum* and *G*. *lamblia* assemblage A or B for each animal species (*Z*_*i*_: mean zoonotic prevalence for species *i*) as shown in Table 3. Studies from India were used when available along with recent literature reviews of *Cryptosporidium* and *Giardia* species (see Table 3). When possible, only data from studies genotyping ≥ 50 animals were included in calculating a mean value to avoid bias from small sample studies (see Table 3). The mean zoonotic prevalence by species was then used to calculate the overall zoonotic fraction, $\hat{Z}$, of parasites shed by non-humans. For the ENV and MST scenarios, $\hat{Z}$, is the weighted average of *Z*_*i*_ values, using the species share of the total animal parasite environmental load as the weight, as follows:

Z^=∑in(Ai×Zi)∑inAi (3)

where *A*_*i*_ is the fraction of parasites shed into the environment from animal source *i* (*i* = cattle, buffalo, sheep, goat, and dog) and *Z*_*i*_ is the mean zoonotic prevalence of parasites shed by animal source *i* (from Table 3).

**Table 3:** Animal host mean prevalence rates (*Z*_*i*_) of shedding zoonotic species infectious-to-humans (i.e., *C*. *parvum* or *G*. *lamblia* assemblage A or B) estimated from published literature and used in Eq 3 to calculate the overall zoonotic fraction ($\hat{Z}$ in Table 1) of *Cryptosporidium* and *Giardia* parasites shed by animals in Puri District able to infect humans.

Parameter	Description	Point Est. (%)	*n* studies	Source
*Zc*_*cattle*_	Cattle prevalence *C*. *parvum*	55	27	[30–35]
*Zc*_*buffalo*_	Buffalo prevalence *C*. *parvum*	49	4	[33, 35]
*Zc*_*sheep*_	Sheep prevalence *C*. *parvum*	35	12	[35]
*Zc*_*goat*_	Goat prevalence *C*. *parvum*	51	6 ^*b*^	[35]
*Zc*_*dog*_	Dog prevalence *C*. *parvum*	18	5 ^*b*^	[36]
*Zg*_*cattle*_	Cattle prevalence *G*. *lamblia* assemblage A or B	19	10	[37]
*Zg*_*buffalo*_	Buffalo prevalence *G*. *lamblia* assemblage A or B	90	3	[37, 38, 56]
*Zg*_*sheep*_	Sheep prevalence *G*. *lamblia* assemblage A or B	5	2	[37]
*Zg*_*goat*_	Goat prevalence *G*. *lamblia* assemblage A or B	5	5 ^*b*^	[37]
*Zg*_*dog*_	Dog prevalence *G*. *lamblia* assemblage A or B	36	8	[37]

^*b*^ No studies with *n* ≥ 50 found so studies with < 50 sample size used.

#### Daily volume of tubewell water consumed and ingested dose

Water ingestion rates are reported to vary by region and by age, with older children and those living in tropical climates (such as Odisha) ingesting more water compared to younger children and those in temperate climates [40, 41]. We did not measure water ingestion rates in our study population. Thus, we used upper estimates of water ingestion from studies in temperate climates that stratified ingestion rates by age [40], and estimates from a study in India (mean of 1.39 L day^-1^ for < 5 children) which monitored direct intake of drinking water over one calendar year and summarized results for children < 5 [41]. Combining these literature values, we represented the daily volume of tubewell water drunk by children by a uniform distribution (min = 0.893 L day^-1^, max = 1.39 L day^-1^) as age distribution data for Puri District < 5 years old was lacking. To account for the effects of boiling water (the only point of use treatment method used by the study population) on parasite viability, we used WHO reported [39] 6 log_10_ reduction values for *Cryptosporidium* and *Giardia* from boiling (i.e. 6 log10 = 99.9999% reduction) and used household survey data collected from study households (n = 355) to estimate the fraction of households that boiled their drinking water (9% and 16% of deep and shallow tubewell users, respectively, reported boiling; see Table 1, *B*, boiling rates) (Appendix B.5 in Supporting Information).

The full equation used to estimate the dose, *d*, of the number of pathogens ingested per day per individual able to cause an infection in humans is:

$$d=\frac{C}{20}\times \frac{1}{MR}\times V\times Sp\times B\times \frac{1}{LR}\times L$$ (4)

where *C* (# 20 L^-1^) is a random sample from the gamma distribution describing the observed concentration of parasites (by year, tubewell type, and protozoa species) adjusted for non-detects, *MR* is the method recovery fraction determined by spiking trials, *V* is the fraction of viable parasites able to cause host infection, *Sp* is the fraction of parasites in the dose which are a species infectious-to-humans, *LR* is the reduction of parasites from boiling (1 if no boiling occurred), *B* is a dichotomous term that indicates if the household boiled (i.e. yes or no), and *L* is the volume of water ingested in liters per day.

### Risk characterization

For children drinking protozoa-contaminated tubewell water in our study population, we estimated the risk of infection for an individual child over the course of a single day (individual risk), accounting for boiling and other factors, and compared the expected risk for different households (i.e., deep *vs*. shallow tubewell users), years (i.e. 2012–2013) and protozoa (i.e. *Cryptosporidium vs*. *Giardia*), to the tolerable level of microbial risk from drinking water for a day (1 in 1,000,000) in the United States [57]. To estimate individual risk of infection for an average child from drinking tubewell water, under the local probability of water treatment via boiling, we produced risk plots for each pathogen and tubewell type and each year using the output from the QMRA model (i.e. probability of infection from drinking tubewell water for one day, see Fig 1). Plots for each scenario were generated from 10,000 Monte Carlo simulations of a single day of exposure to tubewell water for an individual.

### SIR

To estimate the waterborne child diarrhea disease burden in the study population attributable to *Cryptosporidium* and *Giardia* protozoal contamination of tubewell drinking water, we used an SIR model coupled with the QMRA model, above, to longitudinally track the daily infection status of each individual child in our study population over time (Fig 2), assigning each to a tubewell type (*TW*) and to boiling (*B*) before drinking as a function of the household fractions for each practice in the population (see *TW* and *B* in Table 1). SIR models track the daily infectious status of individuals in three states; susceptible to acquiring an infection when exposed (S), infected from an exposure event (I), and recovered from an infection event with some degree of immunity (R). In our SIR model, S means a child is susceptible to infection from *Cryptosporidium* or *Giardia*, I means a child is infected from *Cryptosporidium* or *Giardia* and has developed diarrheic symptoms (children who are infected, but do not develop diarrheic symptoms remain in their current state), and R is a child who has recovered from a diarrheic infection from *Cryptosporidium* or *Giardia* and is immune to infection for the moment. To align with the three-month monsoon period when tubewells were sampled in 2012 and 2013, the SIR model was run on a daily time step for 98 days each year (allowing for eight days of model warm-up to establish baseline distributions of SIR states), such that for a given day, each child was in one of the three states, with each child’s state being independent of other children.

To determine if a susceptible child transitioned into the infected state on a given day, first the QMRA model was used to assign a probability of infection, *P(i)*, modeled as a Bernoulli variable (1 = infection, 0 = no infection), to each child. If *P(i)* = 1, the probability that the infection resulted in a diarrheic symptom (*P(s)*) was modeled based on published studies of the diarrhea morbidity ratio for children diagnosed with *Cryptosporidium* [45, 47] or *Giardia* [9, 46] in developing countries. These included two large scale, multi-country, case-control studies designed to identify primary pathogens responsible for causing diarrhea in children in developing countries (the GEMS and MAL-ED) and two smaller scale regional studies conducted in settings with inadequate access to clean water and sanitation. Variability in the morbidity ratio across studies was modeled as a Bernoulli process with parameter *p* represented by a uniform distribution between of 0.28–0.68 and 0.49–0.59 for *Cryptosporidium* and *Giardia* respectively (see *P(s)* in Table 1). Our estimates did not account for co-infections of pathogens and assumed no other pathogen besides *Cryptosporidium* or *Giardia* were responsible for causing a diarrhea episode.

Not all cases of symptomatic *Cryptosporidium* and *Giardia* infection persist for the same duration of time. To model this variability, we used a duration of illness parameter (*D*) (see Table 1) fit from published sources. For *Cryptosporidium*, we identified four studies [48–51] of children with data on duration of illness (i.e. active shedding of parasites) that allowed fitting this data to a statistical distribution (Fig D in Supporting Information). However, we were unable to find similar information for *Giardia* and therefore used previously published values [17] based on outbreak data to estimate duration of infection for *Giardia*. From these studies, the mean duration of cryptosporidiosis and giardiasis is 10 (IQR 3–14) and 11 (IQR 6–14) days respectively. Once the illness event ended a child was assumed to transition into the recovered state and be temporarily immune to reinfection for a duration of seven days post-infection [17].

Only children in the susceptible state could transition into the infected state and only if their infection resulted in diarrheic symptoms (i.e. both *P(i)* and *P(s)* equal to 1 in the model); susceptible children who were infected without symptoms (*P(i)* = 1 but *P(s)* = 0) remained in the susceptible state, while children in the infected and recovered states were protected from infection in the model. The main parameters of the SIR model, their values and distributions, and sources of data are summarized in Table 1.

Using results from the SIR model, we estimated the diarrheal disease burden associated with drinking tubewell water during each monsoon season (mid-June to mid-September 2012 and 2013) for the population of < 5 children in the Sanitation Trial (population burden) and compared modeling estimates to observed levels of child diarrhea measured in the Sanitation Trial over the same periods. All-cause child diarrhea prevalence in the Odisha Sanitation Trial was measured quarterly as the 7-day recall diarrhea period prevalence reported at a single quarterly surveillance visit for each child in the study population. Thus, to properly compare our QMRA-SIR modeled daily average prevalence over the monsoon period simulation (mid-June to mid-September) to the Trial’s observed 7-day recall period prevalence on a single day during each monsoon season, we sampled the simulated child population and their daily diarrhea prevalence time series over the 90-day monsoon period as they were sampled in the Trial’s surveillance. The tubewell contamination data used in the simulation modeling was collected over the same 3-month period in which each of the 3,385 enrolled Trial children were monitored for diarrhea once, on a random day, using caregiver self-report and recall.

To account for caregiver recall bias when sampling the modeled results, we used a reduced symptom recall correction variable (*Re* in Table 1) to derive each child’s 7-day diarrhea period prevalence status. As there was no study specific information on caregiver recall bias, we used results from a study in neighboring Bangladesh in which children < 5 were followed for three years on a weekly basis and accuracy of caregiver recall of diarrhea assessed [52]. In the Bangladesh study, recall varied between 12% and 52% three to six days after onset of diarrhea. Therefore, we modeled recall as a Bernoulli process, in which recall was 100% on the first two recall days (i.e. *p* = 1), and between 12% and 52% for each day of the remaining five recall days (i.e. *p* was a random uniform variable with values between 0.12 and 0.52). To account for surveillance visits occurring on a random day, a total of 1,000 data sampling simulations were run for each year. Further details are provided in Appendix C in Supporting Information.

### Sensitivity analysis

To estimate how sensitive the model estimated 7-day recall predicted child diarrhea prevalence was to assumptions in the QMRA and SIR models, we ran a global sensitivity analysis with parameters considered in the HUM scenario. Specifically, we employed a density based approach previously described [58]. Briefly, the sensitivity analysis used the cumulative distribution function (CDF) of the QMRA-SIR model output as the primary input and compared an unconditional CDF to a conditional CDF for a given parameter. The unconditional CDF was approximated by evaluating the output of the QMRA-SIR models over the entire parameter space by varying all inputs simultaneously (i.e. output is not conditional on a particular parameter value). The conditional CDF was approximated by holding the parameter of interest constant while varying all other parameters (i.e. output is conditional on the fixed parameter value). Using the Kolmogorov-Smirnov two-sample statistic, a sensitivity index was calculated ranging from 0–1, where an index closer to one indicated the QMRA-SIR model output was more sensitive to that parameter (i.e. the conditional CDF diverged further from the unconditional CDF). To set the bounds of parameter space in the sensitivity analysis, minimum and maximum values were used for QMRA-SIR parameters represented by a distribution of values, while model sensitivity to parameters represented deterministically (i.e. with a single value as with immunity of seven days) was assessed by halving and doubling the parameter value. Uncertainty around the sensitivity index was assessed with bootstrapping [58]. Separate sensitivity analyses were conducted for *Cryptosporidium* and *Giardia* for each sample year (2012 and 2013) and each tubewell type (DTW and STW), resulting in eight sensitivity analyses. To summarize the overall effect for each parasite, we averaged the upper, median, and lower sensitivity indices from the bootstrapping analysis for each parasite across years and tubewell types.

## Results

### Individual risk

**Fig 3:**
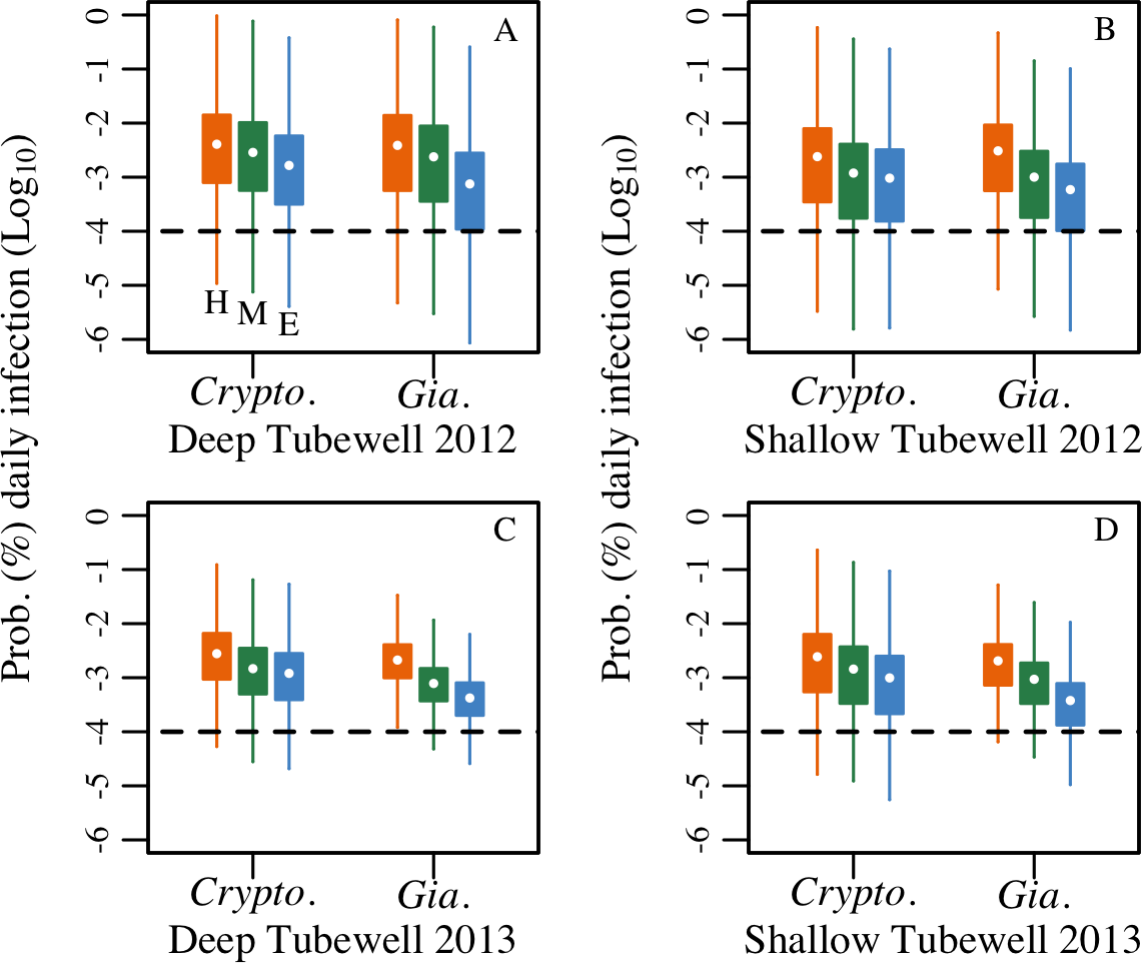
Boxplots of daily risk of *Cryptosporidium* and *Giardia* infection. Daily risk is for an individual child drinking deep and shallow tubewell water in 2012 (A & B) and 2013 (C & D), accounting for boiling rates (9% and 16% by deep and shallow tubewell users respectively), under different scenarios representing the fraction of parasites infectious-to-humans (HUM = H, MST = M, ENV = E). US EPA tolerable daily risk of infection from drinking water is denoted with the dashed line at log_10_ (0.0001%).

Boxplots of daily risk are shown in Fig 3 in log_10_ scale and reveal the mean risk of waterborne protozoal infection per day, accounting for boiling rates, varied between 0.06% and 1.5% (between about 1/1000 and 15/1000 children infected daily) (see Fig F for risk profiles in S1 Supporting Information). Comparing deep and shallow groundwater sources, years, pathogens, and human pathogenicity scenarios (HUM, ENV, MST), ingesting *Cryptosporidium* from deep tubewells in 2012 carried the highest level of daily infection risk (1.5%, 0.7%, 1.2% under the HUM, ENV, and MST scenarios, respectively). The lowest estimated levels of risk were from ingesting *Giardia* in deep and shallow tubewells during 2013 under the ENV scenario (0.063%). A Kolmogorov-Smirnov two-sample test for risk profiles shown in Fig F in Supporting Information indicated a trend that the HUM scenarios were significantly different from the ENV scenario for a given year, tubewell type, and pathogen (see Tables F & G for Kolmogorov-Smirnov two-sample test statistics in S1 Supporting Information)

### Population level risk

**Fig 4:**
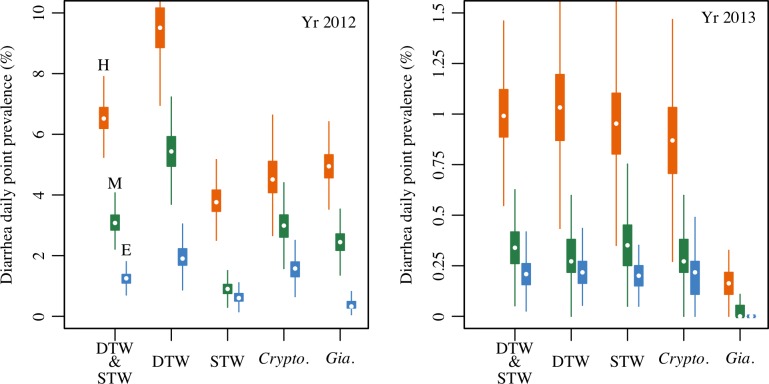
Boxplots of simulated diarrhea daily point prevalence (% of children). Prevalence is shown for the study population during the 90-day monsoon season estimated from 1,000 simulations of the quantitative microbial risk assessment and susceptible-infected-recovered models. Results shown for deep (DTW) and shallow (STW) tubewell users (either infection), and for *Cryptosporidium* (*Crypto*.) and *Giardia* (*Gia*.) infections (either tubewell type) for the monsoon season in 2012 and 2013, under different scenarios used to assign the fraction of parasites infectious-to-humans (HUM = H: orange, MST = M: green, ENV = E: blue). Combined DTW & STW results are for an average child in a tubewell using household, accounting for rates of deep (48%) and shallow (52%) tubewell usage in the study population. Boiling rates (9% and 16% for deep and shallow tubewell users respectively) are accounted for in all results. Different vertical scales for 2012 and 2013 plots highlight the upper limits of burden between years.

Estimated child diarrhea daily point prevalence is shown in Fig 4 and reveals a wide range of prevalence, from a median as high as 6.5% to as low as < 1% at the population level considering estimated levels of water treatment by boiling. Across all tubewell types and years, assuming all parasites were shed from humans (HUM) increased diarrhea prevalence estimates by two-fold or more, compared to using fractions based on environmental parasite loading estimates (ENV) for Puri District (see Table H in S1 Supporting Information). Comparing years, 2012 had a much higher estimated prevalence of diarrhea compared to 2013 under all scenarios. In 2012, deep tubewell users were estimated to have higher diarrhea prevalence than shallow tubewell users, with *Cryptosporidium* often causing more symptomatic infections than *Giardia* across human pathogenicity scenarios. In 2013, deep and shallow tubewell users were estimated to have similar diarrhea prevalence, with considerably more symptomatic *Cryptosporidium* than *Giardia* infections.

The simulated 7-day recall diarrhea period prevalence from waterborne infections are compared to the all-cause observed levels reported in the Odisha Sanitation Trial during the monsoon season (~12% in 2012 and ~ 9% in 2013) [21] in Table 4. The fraction of the observed all-cause diarrhea during each monsoon season that can be explained by the estimated tubewell drinking water protozoa infections under each human pathogenicity scenario (HUM, MST, ENV) is presented. The results indicate that as much as 65.8% or as little as 2.9% of the all-cause diarrhea burden in < 5 children can be attributed to waterborne infections from *Cryptosporidium* and *Giardia* in tubewell water. Depending on the scenario examined (HUM, MST, ENV), we see a wide range of estimated attributable fractions, but a clear trend that a far greater fraction of diarrhea can be attributed to *Cryptosporidium* and *Giardia* contamination in drinking water during the monsoon season in 2012, when child diarrhea prevalence rates were higher (~12% 7-day recall period) compared to 2013 (~9% 7-day recall period). See Table I in S1 Supporting Information for further details.

**Table 4:** Fraction of the observed 7-day recall child diarrhea period prevalence in the Odisha Sanitation Trial (12% in 2012 and 9% in 2013) estimated to be attributable to drinking tubewell water contaminated with *Cryptosporidium* and *Giardia* under different human pathogenicity scenarios examined (median % and interquartile range (IQR)).

Year	HUM (IQR)	MST (IQR)	ENV (IQR)
2012 monsoon season	65.8 (63.4, 68.2)	31.5 (29.8, 33.2)	12.8 (11.5, 13.9)
2013 monsoon season	13.9 (12.4, 15.3)	4.7 (3.8, 5.6)	2.9 (2.3, 3.4)

### Sensitivity analysis

**Fig 5:**
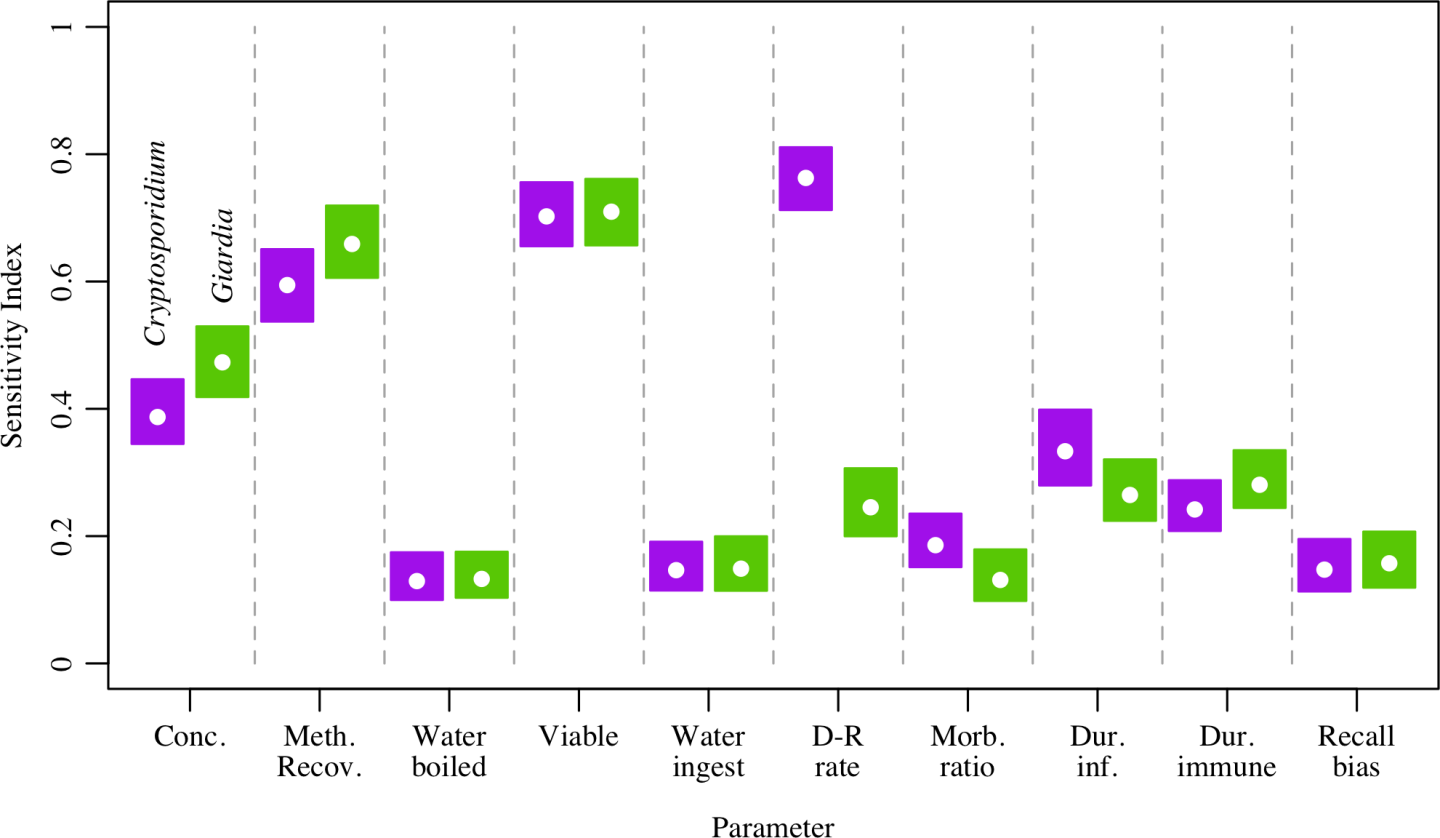
Sensitivity analysis results for *Cryptosporidium* and *Giardia*. Sensitivity analysis for *Cryptosporidium* (purple) and *Giardia* (green) averaged over year and tubewell type showing the sensitivity index of 7-day recall diarrhea period prevalence to each parameter, where a sensitivity index closer to one indicates greater model sensitivity to that parameter.

Results from the sensitivity analysis for the HUM scenario combined by sample year and tubewell type are shown in Fig 5. Of the parameters examined, *Cryptosporidium* 7-day recall diarrhea period prevalence estimates were most sensitive to the rate parameter used in the dose-response model, the fraction of detected parasites assumed to be viable, and the method recovery of parasites from tubewell samples. For *Giardia*, diarrhea estimates were most sensitive to model parameters representing viability, method recovery, and the concentration of *Giardia* observed in tubewell samples. For both parasites, diarrhea estimates were least sensitive to the volume of water ingested, the fraction of the population that treated water by boiling, the morbidity ratio, and recall bias as these parameters had sensitivity indices generally below 0.2.

## Discussion

We coupled QMRA and SIR models to estimate waterborne infection risk and child diarrhea disease burdens attributable to observed *Cryptosporidium* and *Giardia* contamination of tubewell drinking water sources in rural Puri District, Odisha, India and compared model estimated levels to all-cause (all pathogens and pathways) child diarrhea rates measured in the study population. Daily child diarrhea prevalence attributed to infection from *Cryptosporidium* and/or *Giardia* in drinking water was estimated to be between 6.5% and < 1%, depending upon year, tubewell type, and fraction of parasites assumed to be infectious-to-humans. Model-based estimated levels of child diarrhea due to protozoal infections from drinking contaminated tubewell water accounted for as much as 65.8% of the Odisha Sanitation Trial all-cause child diarrhea disease burden measured in the study population (12% and 9% 7-day recall period prevalence, respectively, in 2012 and 2013 monsoon season). While the human pathogenicity scenarios tested in this study demonstrated there was considerable uncertainly around the attributable fraction of observed diarrhea in the Trial from drinking water contaminated with *Cryptosporidium* and *Giardia*, it is likely that the portion of parasites infectious-to-humans lies between the HUM and MST scenarios. Our research shows the usefulness of coupling QMRA-SIR models with field data to estimate the contribution of different pathogens and transmission pathways to diarrheal disease burdens and health impacts associated with targeted water, sanitation, and hygiene interventions.

Our QMRA estimates are the first to estimate waterborne *Cryptosporidium* and *Giardia* infection risk from drinking tubewell water in India and show the microbiological quality of water investigated in this study is unsafe for drinking. Across tubewell types, years, and scenarios, the lowest *daily* estimated additive risk for *Cryptosporidium* and *Giardia*, occurring under the ENV scenario for shallow tubewells in 2013 (0.3% or 3 new infections in 1,000 children per day), exceeded the acceptable limits for *annual* infection risk from daily exposure via drinking water set by the US EPA (0.01% or 1 in 10,000 people) [59]. By comparison, the estimated mean daily risk of infection from *Cryptosporidium* or *Giardia* in this analysis (0.06% - 1.5%) is within the drinking water daily infection risk range reported for *Cryptosporidium* and *Giardia* in groundwater wells in Mexico (0.5–8.4% and 1.9–17% respectively) [19], but below the infection risk reported for *Giardia* in tubewell water used for drinking in Nepal (17%) [60], and for *Giardia* in children in Brazil drinking from tubewells (9.1–29%) [20]. Nevertheless, a mean daily risk as high as 1.5% and as low as 0.06% represents a significant public health threat for children < 5 years old using tubewells with similar levels of protozoal contamination for drinking without proper disinfection or treatment prior to consumption. Aside from the expectation that risk differs geographically, temporally, and between age groups, comparison of estimated risks between QMRA studies is problematic due to different model assumptions and sources of data. However, our QMRA model included important assumptions, such as adjusting for the viability of parasites and accounting for the fraction protozoa species infectious-to-humans, not typically included in QMRA models of *Cryptosporidium* or *Giardia*.

We estimated that of the observed child diarrheal disease burden in the Odisha Sanitation Trial study population, somewhere between 65.8% and 31.5% in 2012, and between 13.9% and 4.7% in 2013, was caused by *Cryptosporidium* and *Giardia* contaminated drinking water, based on the HUM and MST scenarios (Table 4). While the ENV scenario suggested much less and as little as 2.9%, this scenario is unlikely as additional research in the study region found a strong relationship between the level of *Cryptosporidium* and *Giardia* contamination in tubewells and spatial proximity to household latrines, indicating a greater likelihood that parasites originated from humans [61]. Assuming these ranges are correct, we could expect consistent and effective household water treatment (HWT) [62] to reduce diarrheal burdens by up to these fractions and likely more as other waterborne child diarrheal pathogens, including pathogenic *Escherichia coli*, rotavirus, adenovirus, and *Vibrio cholera* were also detected in the tubewell drinking water source samples in the Sanitation Trial [55] in addition to *Cryptosporidium* and *Giardia*. Previous work evaluating the effect of HWT on diarrhea prevalence has had mixed results, with some studies finding as much as 40% reduction in diarrhea and others finding no effect [63, 64]. A randomized control trial evaluating the effectiveness of chlorine for HWT in Puri District recently reported diarrhea prevalence in children < 5 years dropped by roughly 30% (1.23 *vs*. 1.78%) in households verified to use chlorination compared to those who did not [65], further indicating waterborne transmission of diarrheal disease is an important pathway in Puri District. Therefore, until tubewells in Puri District and similar low-income settings can be assured to be microbiologically safe, consistent and effective HWT (chlorination may not destroy all *Cryptosporidium* and *Giardia* parasites under typical concentrations and exposure times) may play an important role in reducing child diarrhea.

Both our model-based estimate of diarrheal disease and the observed levels of all-cause diarrhea in the Trial were higher during the 2012 monsoon season compared to 2013. For our model-based estimates, the differences between years is purely a function of the difference in observed concentration, with 2012 having higher concentration of parasites compared to 2013, as all other parameter distributions/values in the model were consistent across years. Previous analysis of observed levels of all-cause diarrhea in the Trial also attributed differences in diarrhea prevalence between years to levels of tubewell contamination, but also noted a rainfall effect and found an association between lower all-cause diarrhea in 2013 and increased rainfall in one region of the Trial [55]. As mechanisms at work to distribute pathogens in the environment and result in exposure for a host across different years are likely site specific and interacting, a surveillance program would ultimately be needed to gather data and better characterize how changing environmental and host-pathogen demographics interact with each other over annual and other time scales.

Our study has shown that the fraction of parasites in drinking water that are from humans *vs*. non-human animal hosts and that are species infectious-to-humans have important implications for infection risk and diarrhea disease burden estimates. Determining whether *Cryptosporidium* and *Giardia* detected in water samples are infectious-to-humans, however, is difficult. Molecular characterization, such as PCR, may only amplify one species in a sample of mixed species [66], while visual identification at the species levels is not considered valid [67]. To navigate these limitations, it has been suggested that studies of zoonotic diarrheal pathogens might use additional information of the environmental pathways that contaminate tubewell water, such as hydraulic connections from surface to groundwater, combined with information on the spatial distribution of human and animal feces around tubewells to help clarify the relative likelihood of human *vs*. non-human water contamination [61]. Another limitation of our study, specific to the MST and ENV scenarios examining prevalence of zoonotic *Cryptosporidium* and *Giardia* in livestock and domestic animals, was a lack of region specific data. Using studies from India, when available, combined with data from literature reviews to estimate the zoonotic fraction term in our QMRA-SIR models may not have adequately represented the cultural, geographic, and socioeconomic factors that relate to levels of zoonotic parasites in Puri District. However, as numerous factors interact to produce levels of zoonotic pathogens in a population, and as these factors may be spatially and temporally dynamic, a regional surveillance program would ultimately be needed to estimate the fraction of livestock and domestic animals shedding zoonotic parasites. In the meantime, using a range of values, preferably generated from local data sources, for human *vs*. non-human parasites is recommended to realize upper and lower limits of risk.

While we had site-specific pathogen concentrations in source water and method recovery data for our QMRA modeling, we lacked specific information on the viability of the detected *Cryptosporidium* and *Giardia* parasites. Currently, common methods used to detect *Cryptosporidium* and *Giardia* in water do not assess if parasites are able to cause infection [68], and doing so requires additional time, expense, and expertise [67]. Those studies that have investigated viability of parasites in water tend to use viable dye assays or morphology [26, 69], but little information is available for field studies using animal infectivity or culture-based assays (a gold standard) [67], with no data from low-income settings found in our searches. Our assumption taken from a study in North America that infectivity was typically less than 50% for *Cryptosporidium* and less than 25% for *Giardia* reduced our risk estimates by more than half and may have biased our estimates of risk downwards and not fully represented the processes occurring in our study region that increase or inhibit parasite viability. Results from the sensitivity analysis indicate that assumptions about viability are an important factor contributing to variability in our QMRA-SIR model estimates of diarrhea (especially for *Giardia*). Additional information on the viability of parasites detected in drinking water, especially in low-income settings, would help to improve the understanding of risks associated with drinking water contaminated with *Cryptosporidium* and *Giardia*.

The novelty of our research was to couple QMRA estimates of waterborne pathogen infection risk with SIR modeling to estimate the contribution of waterborne infections to childhood diarrhea disease burdens in a low-income setting. Often diarrheal disease risk is shared among multiple transmission pathways, each requiring a different intervention strategy, and among multiple pathogens, often requiring further design considerations for a given WASH intervention. Additionally, the magnitude of impacts on health for a given intervention is likely to differ across settings depending on the major transmission pathways and pathogens of concern in each setting. The modeling methods developed here provide a new approach to assist in more effective selection and targeting of interventions to maximize health impacts on diarrheal disease at the local level. However, using model results without field observations makes the approach less useful. Having data on exposure (drinking water quality) and outcomes (child diarrhea rates) for the same population over the same time period, allowed us to both construct the QMRA-SIR modeling and compare the results against observed outcomes. More field, monitoring, and evaluation studies for diarrheal disease should aim to do this.

While waterborne transmission is clearly a contributor of diarrheal disease burdens and while outbreak data suggest *Cryptosporidium* and *Giardia* are important etiological agents of waterborne disease worldwide [5], there are other important diarrheal transmission pathways and pathogens to consider. In our setting, analyses of water samples from Puri District during 2012 and 2013 detected four other diarrheal pathogens (pathogenic *Escherichia coli*, rotavirus, adenovirus, and *Vibrio cholera*) in these same tubewells at similar detection rates to *Cryptosporidium* and *Giardia* [55]. Additionally, improved sanitation and hygiene is also lacking in Puri District and animal contact is frequent. These conditions make transmission from direct contact with human and non-human feces a likely additional cause of diarrhea. While we demonstrated that tubewells contaminated with *Cryptosporidium* and *Giardia* had a high potential of being a source of diarrhea in Puri District, coupling waterborne, foodborne, handborne, and other disease transmission models that incorporate environmental pathogen transport processes, such as transport mediated by rainfall, and consider multiple pathogens would be an important step to more fully understand diarrheal disease etiology, pathways of transmission, and risk factors in low-income settings. Additionally, the use of sensitive diagnostic methods able to detect the relativity low, but public health relevant concentrations of *Cryptosporidium* and *Giardia* used in this study should be a goal for water quality research programs going forward to better characterize the risks associated with drinking water contamination. In the meantime, diarrheal disease burdens in Puri District and other similar settings may persist despite improvements in sanitation and hygiene, unless drinking water is made safe and reliable at the source or through effective household water treatment.

## Supporting information

S1 Supporting Information Detailed description of modeling inputs and outputs. (DOCX)
