# Supplementary material for: Estimating Cryptosporidium and Giardia disease burdens for children drinking untreated groundwater in a rural population in India
Source: PLoS Negl Trop Dis. 2018 Jan 29;12(1):e0006231. doi: 10.1371/journal.pntd.0006231 (PMC5805363; doi:10.1371/journal.pntd.0006231)
Supplement: S1 Supporting Information — (DOCX) [file pntd.0006231.s001.docx]

**S1 Supporting Information**

Estimating *Cryptosporidium* and *Giardia* disease burdens for children drinking untreated groundwater in a rural population in India

Miles E. Daniels ^1, 2 ¶*^, Woutrina A. Smith ^1 ¶^, and Marion W. Jenkins ^3 ¶^

^1^ Department of Veterinary Medicine and Epidemiology, School of Veterinary Medicine, University of California, Davis, United States of America

^2^ Department of Institute of Marine Sciences, University of California, Santa Cruz, United States of America, Affiliated with: Fisheries Ecology Division, Southwest Fisheries Science Center, National Marine Fisheries Service, National Oceanic and Atmospheric Administration, United States of America

^3^ Department of Civil and Environmental Engineering, University of California, Davis, United States of America

***** Corresponding author

E-mail: [miles.daniels@ucsc.edu](mailto:miles.daniels@ucsc.edu) (MED)

¶ These authors contributed equally to this work

1. **Dose-response**

For both *Cryptosporidium* and *Giardia*, an exponential distribution has been determined to fit experimental feeding trial best [1].To estimate appropriate dose-response parameters, a review of relevant literature was used. To characterize the uncertainty around these estimates, associated with differences in parasite virulence, study design, host immunity, and other factors, a triangular distribution was used, where the minimum and maximum values of the distribution were set the minimum and maximum value in published sources with the mode set to the maximum likelihood estimate. The Exponential distribution is:

| $P_{i}=1-e^{-kn}$ | (S1) |
| --- | --- |

where *P_i_* is the probability of infection given the number of parasites ingested (*n*) and *k,* the rate parameter, where the closer *k* is to one, the greater the chance of infection for a given *n*.

From the literature, *Cryptosporidium* was found to have a rate parameter between 0.00021 and 0.05 and between 0.0097 and 0.0358 for *Giardia* [2-4]*.* Therefore, a triangular distribution T(0.00021, 0.0042, 0.05) was used for *Cryptosporidium* and T(0.009798, 0.0198, 0.03582) was used for *Giardia*. Fig. A shows the cumulative density function of the dose-response function for both *Cryptosporidium* and *Giardia*.

**Fig A:** Cumulative density function of exponential distribution for *Cryptosporidium* (solid line) and *Giardia* (dashed line) showing probability of infection as a function of parasites ingested with upper and lower limits shown in shaded region (light for *Cryptosporidium* and dark for *Giardia*).

1. **Exposure assessment**

To evaluate how different exposure types, such as drinking shallow versus deep tubewell water, influences the risk of illness, multiple scenarios were simulated. For example, if we were to assess the risk of drinking tubewell water contaminated with *Cryptosporidium*, we would consider characteristics of the scenario such as: quality of water ingested, prior treatment of water, and volume of water ingested. Each characteristic was a separate parameter in the model described by a statistical distribution or point estimate to account for the variability inherent in one scenario to the next and the uncertainty around each parameter estimate.

- 1. **Adjusting non-detects**

To account for the lower limit of detection of *Cryptosporidium* oocysts and *Giardia* cysts using IMS-DFA for detection and enumeration, we replaced non-detect samples with ½ the value of the lower limit of detection of the assay (i.e. 1 parasite per DFA slide well). Concentration of oocysts/cysts in each non-detect water sample were calculated as follows:

| $C=\left\{ \left[ \frac{{PU}_{v}}{{PA}_{v}} \times IMS_{frac} \times\frac{LOD}{2} \right] \times\left[ \frac{\frac{{RA}_{v}}{{RU}_{v}}}{S_{v}} \right] \right\}\times20$ | (S2) |
| --- | --- |

where *C* is *Cryptosporidium* or *Giardia* concentration (# 20 L^-1^), ${PU}_{v}$ is pellet volume used (mL) and ${PA}_{v}$ is the pellet available of the water sample after centrifugation, *IMS_frac_* is the fraction of IMS material applied to a slide (1/2), *LOD* is the limit of detection for parasites counted on a slide (i.e. 1), ${RU}_{v}$ is the retentate volume used (mL) and ${RA}_{v}$ is the retentate volume available (mL) after Fresenius filtration of the total water samples volume $S_{v}$ (L).

- 1. **Assigning distribution of *Cryptosporidium* and *Giardia* concentrations in tubewells**

Across the 2012 and 2013 monsoon sampling seasons, there were 110 deep tubewell samples and 96 shallow tubewells samples. A gamma distribution, with the parameterization of a shape and scale (mean/shape), was used to fit the observational data (# 20 L^-1^), as concentrations were non-negative and continuous. To fit distributions to the data, three independent chains of Metropolis Markov Chain Monte Carlo (MCMC) simulations (50,000 iterations each) with non-informative priors for the gamma distribution (shape = uniform [0, 40] and mean = uniform [0, 40]) were ran [5]. All analysis was performed in the R modeling environment [6]. Model convergence was determined using the Gelman and Rubin diagnostic in the BOA package for R [7]. Table A shows the posterior parameters values for the gamma distribution for each protozoa, water source, and year in addition to the mean concentration from the fitted gamma distribution.

**Table A:** Posterior gamma scale and shape values for each protozoa, water source, and year, with mean concentration.

| Protozoa, water source, and year | Posterior gamma scale parameter | Posterior gamma shape parameter | Fitted mean conc.  (# 20 L^-1^) |
| --- | --- | --- | --- |
| *Cryptosporidium* |  |  |  |
| Deep2012 | 16.633 | 0.8974 | 13 |
| Deep2013 | 0.301 | 18.316 | 6 |
| Shallow 2012 | 5.845 | 1.437 | 6 |
| Shallow 2013 | 0.282 | 19.667 | 6 |
| *Giardia* |  |  |  |
| Deep2012 | 41.289 | 0.579 | 23 |
| Deep2013 | 0.179 | 29.569 | 5 |
| Shallow 2012 | 12.128 | 1.173 | 14 |
| Shallow 2013 | 0.362 | 15.601 | 6 |

- 1. **Assigning distribution for method recovery uncertainty in parasite detection**

Spiking studies of *Cryptosporidium* and *Giardia* in water have shown that method recovery efficiency can be highly variable [8]. This variability can have important consequences for QMRA when ignored, and result in a 100-times underestimation of concentration levels under certain conditions (i.e. when method recovery is low under-estimation is high). To estimate method recovery for this QMRA analysis, Approach II outlined in Petterson, Signor (8) was used. This approach adjusts observed concentrations using method recovery data unpaired from raw counts (i.e. water sampled at sites and spikes are different).

Fig. B shows the recovery data from 11 spiking trials done during the 2012 and 2013 monsoon sampling seasons. All spikes were conducted in India. Briefly, each spike consisted of adding 200 *Cryptosporidium* oocysts and *Giardia* cysts to 20 liters of DI water. Spikes were processed identically to tubewell samples to quantify the number of parasites recovered (i.e. Fresenius filtration followed by Immunomagnetic separation and direct fluorescence antibody assays).

**Fig B:** Method recovery of *Cryptosporidium* and *Giardia* spiked into DI water. Both individual data (points) and mean recovery (dashed lines) are shown (Mean *Cryptosporidium* = 55%, *Giardia* = 34%).

For each set of pathogen data, a beta-binomial distribution was fit to the recovery data. Briefly, each parasite spiked into a water sample followed a binomial distribution, with a probability *p* of being recovered and detected and probability of 1-*p* of being missed, with each spike consisting of 200 independent trials. The beta distribution with parameters (α and β) described the variability between spiking trials. To fit distributions to the data, three independent chains of Metropolis Markov Chain Monte Carlo (MCMC) simulations (50,000 iterations each) with non-informative priors for the beta distribution (α = uniform [0.5, 20] and β = uniform [0.5, 20]) were ran [5]. All analysis was performed in the R modeling environment [6]. Model convergence was determined using the Gelman and Rubin diagnostic in the BOA package for R [7]. To account for the variability between spiking trials, 1,000 random samples of the α and β parameters posterior distribution were randomly sampled to obtain the distribution of method recovery for each parasite (Fig. C). Model fitting results indicate *Cryptosporidium* recovery was distributed beta-binomial (1.9, 1.9) and *Giardia* recovery was distributed beta-binomial (3, 6).

**Fig C:** Posterior distributions for the α and β parameters of the beta-binomial distribution and the method recovery (expressed as a fraction) distribution for both *Cryptosporidium* and *Giardia*.

To adjust the observed concentrations of *Cryptosporidium* and *Giardia* for method recovery the following formula was used each model iteration:

| $C_{recover}=C_{obs} \times\frac{1}{MR}$ | (S3) |
| --- | --- |

where *C_recover_* is the adjusted concentration value, *C_obs_*is the observed concentration calculated from the IMS-DFA results after adjusting for non-detects, and *MR* is a random sample of method recovery from the beta-binomial distribution specific for each protozoal pathogen.

- 1. **Assignment of drinking water source**

Using a baseline survey conducted in the study population in 2010, it was estimated that 82% of the population used tubewell water for drinking purposes, with 52% of those owning and using a shallow tubewell in their home or in their own compound and 48% using deep tubewells. Therefore, these percentages were used to split the simulated population into two groups, those who do or do not use a deep tubewell.

- 1. **Assigning distribution of treatment of drinking water source**

Using the data collected from households (n = 355) while sampling in the 2012 and 2013 monsoon season, it was estimated that ~ 9% of the population who used deep tubewells water for drinking purposes boiled their water and that 16% owning a shallow tubewell boiled their water. Therefore, treating water by boiling was distributed Bernoulli (0.09) in deep tubewells and Bernoulli (0.16) in shallow tubewells. Boiling water was assumed to reduce virulent *Cryptosporidium* and *Giardia* at a 6 log_10_ reduction factor [9]. The formula to adjust concentrations of infectious parasites after boiling is:

| $C_{post}=C_{pre} \times\frac{1}{LR}$ | (S4) |
| --- | --- |

where *C_post_* the adjusted concentration value after boiling, *C_pre_*is the observed concentration calculated from the IMS-DFA results and adjusted for percent recovery and non-detects, and *LR* is a log_10_ reduction factor (i.e. 6 log10 = 99.9999% reduction) from boiling water.

- 1. **Assigning fraction of parasites from livestock and domestic animals versus humans**

As the morphology of human and non-human shed parasites are considered to be indistinguishable from each other and as both human and livestock/domestic animals shed *Cryptosporidium* and *Giardia* in Puri District[10], we employed three different strategies to differentiate if *Cryptosporidium* and *Giardia* parasites in tubewell samples were of human origin or of livestock/domestic animal origin.

- - 1. **Human (HUM) Method**

To explore a worst-case scenario, we assumed all of the detected parasites were shed from humans and therefore infectious-to-humans.

- - 1. **Environmental Loading (ENV) Method**

For the ENV scenario, we used four primary pieces of information to calculate the environmental loading rate of *Cryptosporidium* and *Giardia* by humans in our study region: parasite prevalence, parasite shedding rate, host population demographics, and host fecal production rates. Prevalence was estimated from a cross sectional study in a rural Indian village that lacked access to improved sanitation, where fecal samples from 78 people were repeatedly collected over a period of a month on alternate days with results presented as point estimates across four age categories (< 5 years, 5-10 years, 11-15 years, and > 15 years old) [11]. Shedding rates of *Cryptosporidium* and *Giardia* were estimated as the arithmetic mean from a cross sectional study in Puri District from 85 people [10] and was assumed to be constant across age categories. The total population of Puri District was obtained from a census report of Odisha in 2010 [12] and the age distribution of Puri was assumed to follow the national distribution [13]. Fecal production rates were obtained from a review focused on assessing on-site sanitation of feces in developing countries [14] with the variability in feces production described by a triangular distribution. All information was stratified into the four age categories described above. See Table B for specific values.

**Table B:** Parameters used to estimate environmental loading characteristics of *Cryptosporidium* and *Giardia* from humans in Puri District

| Parameter | Description | Value | Units | Source |
| --- | --- | --- | --- | --- |
| *NH* | Human population in rural Puri District (Point estimate) | 1,298,654 | # | [12] |
| *FH* _< 5 yrs_ | < 5 yrs population | 9.8 | % | [13] |
| *FH* _5-10 yrs_ | 5-10 yrs population | 10 | % | [13] |
| *FH* _11-15 yrs_ | 11-15 yrs population | 9.9 | % | [13] |
| *FH* _> 15 yrs_ | >15 yrs population | 70.3 | % | [13] |
| *PH* _< 5 yrs_ | < 5 yrs prevalence *Cryptosporidium* | 45 | % | [11] |
| *PH* _5-10 yrs_ | 5-10 yrs prevalence *Cryptosporidium* | 50 | % | [11] |
| *PH* _11-15 yrs_ | 11-15 yrs prevalence *Cryptosporidium* | 60 | % | [11] |
| *PH* _> 15 yrs_ | >15 yrs prevalence *Cryptosporidium* | 30 | % | [11] |
| *PH* _< 5 yrs_ | < 5 yrs prevalence *Giardia* | 75 | % | [11] |
| *PH* _5-10 yrs_ | 5-10 yrs prevalence *Giardia* | 65 | % | [11] |
| *PH* _11-15 yrs_ | 11-15 yrs prevalence *Giardia* | 60 | % | [11] |
| *PH* _> 15 yrs_ | >15 yrs prevalence *Giardia* >15 yrs | 30 | % | [11] |
| *MH* _< 5 yrs_ | < 5 yrs fecal production *^a^* | T (75, 224, 374) | g day^-1^ | [14] |
| *MH* _5-10 yrs_ | 5-10 yrs fecal production *^a^* | T (75, 224, 374) | g day^-1^ | [14] |
| *MH* _11-15 yrs_ | 11-15 yrs fecal production *^a^* | T (75, 224, 374) | g day^-1^ | [14] |
| *MH* _> 15 yrs_ | >15 yrs fecal production *^a^* | T (75, 243, 520) | g day^-1^ | [14] |
| *SH* | *Cryptosporidium* Shedding rate *^b^* | 107 | # gram^-1^ | [10] |
| *SH* | *Giardia* Shedding rate *^b^* | 12 | # gram^-1^ | [10] |

*^a^* Triangular distribution has three parameters = T (min, mean, max)

*^b^* calculated as arithmetic mean from samples reported as DFA positive by Daniels et al. 2015

We assumed the remaining non-human fraction of detected parasites was shed by livestock (predominantly cattle, plus buffalo, sheep, and goats) and domestic animals (predominantly dogs) as these species represented the majority of animals in the study region. To calculate environmental loading rates for livestock and domestic animal species, we used four primary prices of information as described previously [10]: parasite prevalence, parasite shedding rate, host population demographics, and host fecal production rates. Host-specific/pathogen-specific prevalence and shedding rates (arithmetic mean) were derived from samples collected in Puri District during 2012, with uncertainty in prevalence estimates represented by a triangular distribution. Population estimates were obtained from unpublished census data of livestock in Puri District and combined with published fecal production rates to estimate total fecal production rates by host-species. Tables C summarizes the data used to estimate parasite loading in Puri District and each host species contribution to the total load. We assumed the probability a parasite detected in a water sample was shed from a particular host species was directly proportional to the estimated contribution of parasites from that host species in Puri District.

**Table C:** Parameters used to estimate environmental loading characteristics of *Cryptosporidium* and *Giardia* from livestock/domestic animals in Puri District

| Parameter | Description | Value | Units | Source |
| --- | --- | --- | --- | --- |
| *NA* | Animal population in rural Puri District | 702,328 | # | [10] |
| *N _cattle_* | Cattle population | 429,397 (61) | # (%) | [10] |
| *N _buffalo_* | Buffalo population | 27,401 (4) | # (%) | [10] |
| *N _sheep_* | Sheep population | 67,466 (9) | # (%) | [10] |
| *N _goat_* | Goat population | 132,717 (19) | # (%) | [10] |
| *N _dog_* | Dog population | 45,347 (7) | # (%) | [10] |
| *PA _cattle_* | Cattle prevalence *Cryptosporidium* *^a^* | T (0, 5, 27) | % | [10] |
| *PA _buffalo_* | Buffalo prevalence *Cryptosporidium* *^a^* | T (0, 5, 25) | % | [10] |
| *PA _sheep_* | Sheep prevalence *Cryptosporidium* *^a^* | T (16, 35, 60) | % | [10] |
| *PA _goat_* | Goat prevalence *Cryptosporidium* *^a^* | T (16, 35, 60) | % | [10] |
| *PA _dog_* | Dog prevalence *Cryptosporidium* *^a^* | T (0, 17, 42) | % | [10] |
| *PA _cattle_* | Cattle prevalence *Giardia* *^a^* | T (20, 40, 64) | % | [10] |
| *PA _buffalo_* | Buffalo prevalence *Giardia* *^a^* | T (0, 9, 31) | % | [10] |
| *PA _sheep_* | Sheep prevalence *Giardia* *^a^* | T (24, 45, 68) | % | [10] |
| *PA _goat_* | Goat prevalence *Giardia* *^a^* | T (4, 15, 39) | % | [10] |
| *PA _dog_* | Dog prevalence *Giardia* *^a^* | T (41, 67, 86) | % | [10] |
| *MA _cattle_* | Cattle fecal production | 38,400 | g day^-1^ | [10] |
| *MA _buffalo_* | Buffalo fecal production | 38,400 | g day^-1^ | [10] |
| *MA _sheep_* | Sheep fecal production | 675 | g day^-1^ | [10] |
| *MA _goat_* | Goat fecal production | 1,664 | g day^-1^ | [10] |
| *MA _dog_* | Dog fecal production | 249 | g day^-1^ | [10] |
| *SH _cattle_* | Cattle shedding rate *Cryptosporidium* *^b^* | 4 | # gram^-1^ | [10] |
| *SH _buffalo_* | Buffalo shedding rate *Cryptosporidium* *^b^* | 1 | # gram^-1^ | [10] |
| *SH _sheep_* | Sheep shedding rate *Cryptosporidium* *^b^* | 1,325 | # gram^-1^ | [10] |
| *SH _goat_* | Goat shedding rate *Cryptosporidium* *^b^* | 29 | # gram^-1^ | [10] |
| *SH _dog_* | Dog shedding rate *Cryptosporidium* *^b^* | 82 | # gram^-1^ | [10] |
| *SH _cattle_* | Cattle shedding rate *Giardia* *^b^* | 844 | # gram^-1^ | [10] |
| *SH _buffalo_* | Buffalo shedding rate *Giardia* *^b^* | 9 | # gram^-1^ | [10] |
| *SH _sheep_* | Sheep shedding rate *Giardia* *^b^* | 482 | # gram^-1^ | [10] |
| *SH _goat_* | Goat shedding rate *Giardia* *^b^* | 4 | # gram^-1^ | [10] |
| *SH _dog_* | Dog shedding rate *Giardia* *^b^* | 3,023 | # gram^-1^ | [10] |

*^a^* Triangular distribution has three parameters = T (min, mean, max)

*^b^* calculated as arithmetic mean from samples reported as DFA positive by Daniels et al. 2015

The following formulas are used to calculate loads of parasites from human and livestock/domestic animals:

| $LH_{age}=\left( NH \times FH_{age} \right) \times PH_{age} \times MH_{age} \times SH_{parasite}$ | (S5) |
| --- | --- |

| $L A_{species}= NA_{species} \times PA_{species} \times MA_{species} \times SA_{species + parasite}$ | (S6) |
| --- | --- |

| $Total {Load}_{parasite}= \sum\begin{aligned} {L H}_{<5yrs}, L H_{5-10yrs}, L H_{11-15yrs}, L H_{>15yrs}, \\ L A_{cattle}, L A_{buffalo}, L A_{sheep}, \\ L A_{goat}, L A_{dog} \end{aligned}$ | (S7) |
| --- | --- |

where *L* is loading rate of parasites (n day^-1^), for humans (*H*) and livestock/domestic animals (*A*), *N* is the population size of individuals, *F* is the fraction of humans in a specific age category, *P* is the prevalence of parasite shedding (%), *M* is the mass loading of fecal material produced (grams individual^-1^ Day^-1^), *S* is the shedding rate of parasites in feces per individual (# gram^-1^), with loading rates calculated for different age categories (*age*) for humans (< 5 yrs, 5-10 yrs, 11-15 yrs, and > 15 yrs) and different species (*species*) for animals (cattle, buffalo, sheep, goat, dog), and the total load of each parasite is the sum of each individual parasite load for each human age category and animal species.

- - 1. **Microbial Source Tracking (MST) Method**

Microbial source tracking (MST) uses molecular methods to detect enteric bacteria specific to a host to identify the likely source of fecal contamination. We used MST data from the same water samples that were screened for *Cryptosporidium* and *Giardia* as a second means of differentiating between human and livestock/domestic animal parasites detected in tubewells. We assumed the probability a parasite detected in a water sample was of human origin was directly proportional to the fraction of water samples positive for human specific MST marker (BacHum) compared to the fraction of water samples positive for either human or livestock/domestic animal MST marker (BacHum + BacCow). Table D below shows the relative fractions of humans versus animal contributions to protozoal tubewell contamination calculated from Odagiri, Schriewer (15) using the following formulas:

| $F_{MST} H={P_{BacHum}}/\left( P_{BacHum}+P_{BacCow} \right)$ | (S8) |
| --- | --- |

| $F_{MST} A={P_{BacCow}}/\left( P_{BacHum}+P_{BacCow} \right)$ | (S9) |
| --- | --- |

where *F_MST_* is the fraction of human (*H*) and animal (*A*) parasites in tubewell samples determined using the MST method, *P* is the prevalence of human specific (*BacHum*) and livestock/domestic animal specific (*BacCow*) MST markers.

**Table D:** Parameters used to estimate the probability of human shed *vs.* livestock/domestic animal shed *Cryptosporidium* and *Giardia* in tubewells in Puri District with the MST method

| Parameter | Description | Value | Units | Source |
| --- | --- | --- | --- | --- |
| *F H _Gov 12_* | Human deep tubewell 2012 | 50 | % | [15] |
| *F H _Priv 12_* | Human shallow tubewell 2012 | 16 | % | [15] |
| *F H _Gov 13_* | Human deep tubewell 2013 | 20 | % | [15] |
| *F H _Priv 13_* | Human shallow tubewell 2013 | 33 | % | [15] |

* Note: livestock/domestic animal fractions are 1- human fractions

- 1. **Assigning fraction of parasites able to cause infection (viability)**

As not all parasites detected in water samples are found to be infectious (i.e. viable) and since we lacked information from our study about the infectivity of detected parasites, we used published data [16, 17] to estimate the fraction of parasites in water samples which are infectious. From model fitting, *Cryptosporidium* viability was distributed beta binomial (1.65, 2.46) and *Giardia* was beta binomial (2.93, 17.4), corresponding to a mean viability percent of 38 and 13% respectively. Concentrations were adjusted stochastically in the model using the reciprocal of the viability estimate.

- 1. **Assigning distribution of volume water ingested per day**

Using published data it is estimated that children will ingest between 0.893 and 1.39 liters of water per day on average [18, 19]. Therefore, ingestion rates of tubewell water are modeled as a uniform distribution with those upper and lower limits.

- 1. **Assigning distribution of symptomatic versus asymptomatic illness (morbidity ratio)**

Some events of parasite infection will not result in symptomatic cases (diarrhea in this study), but rather result in asymptomatic infection. To model this process we used data from studies in children for both *Cryptosporidium* and *Giardia* and estimated the morbidity ratio, i.e. the probability that an infection resulted in a symptomatic case of diarrhea. Sarkar, Ajjampur (20) performed a study in a semi-urban slum area of South India. A total of 176 pre-weaning children were followed until the age of two using PCR to diagnosis *Cryptosporidium* infection combined with self-reported diarrhea. Cordón, Soldan (21) conducted a study of 845 children between the ages of 1-9 in Peru and used PCR for parasite identification combined with self-reported diarrhea. Additionally, we included data from two large scale, multi-country, case-control studies designed to identify primary pathogens responsible for causing diarrhea in children in developing countries (the GEMS and MAL-ED [22, 23]). From these studies the morbidity ratio was distributed Bernoulli, where the *p* parameter was uniform (0.29-0.68) for *Cryptosporidium* and uniform (0.49-0.59) for *Giardia*.

- 1. **Assigning distribution of the duration of illness**

Not all cases of symptomatic *Cryptosporidium* and *Giardia* infection persist for the same duration of time. To model this variability, we used data from published sources. For *Cryptosporidium*, we identified four studies [24-27] of children with enough information to extract duration of illness information and fit this data to a statistical distribution (Fig D). However, we were unable to find similar information for *Giardia* and therefore used values reported by Enger, Nelson (28), who used previously published outbreak data to estimate duration of infection for *Giardia*. From these studies, duration of cryptosporidiosis was distributed gamma (1.1, 9.2) and giardiasis was distributed gamma (3.2, 3.4), representing mean durations of 10 and 11 days respectively.

**Table E:** Parameters from gamma distribution used to estimate duration of illness

| Parameter | Description | Value | Units | Source |
| --- | --- | --- | --- | --- |
| *Giardia _days_* | *Giardia* duration of illness | Gamma (3.2, 3.4) | days | [28] |
| *Crypto. _days_* | *Cryptosporidium* duration of illness | Gamma (1.1, 9.2) | days | [24-27] |

**Fig D:** Gamma distribution fit of duration of illness from *Cryptosporidium* infection from published data sources A[24], B[25], C[26] and D[27].

- 1. **Assigning distribution of the days of immunity**

Immunity is a complex process that is likely dependent on numerous host and location specific factors. Previous studies modeling disease transmission for fecal pathogens have used a 7-day duration for *Giardia* immunity [28]. In our model, individuals previously infected with *Cryptosporidium* or *Giardia* and being symptomatic and recovered are assumed to have a 7-day immunity from that pathogen for simulation purposes.

1. **Risk Characterization**

Risk characterization is the process of combining both dose-response and exposure information to calculate the probability or risk of illness given certain scenarios, such as ingesting contaminated shallow tubewell water.

To predict illness from ingesting parasites from contaminated tubewell water, a disease transmission simulation approach was used to model the probability that an individual (*i*) had a symptomatic infection on a given day (*j*). To simulate this process, a matrix was used (Fig E). For each *i*^th^ individual on day *j*, a parameter set was used to determine, how many parasites an individual ingested, if the ingested number caused an infection, followed by if symptomatic infection occurred, and for how long symptomatic infection occurred, and if the individual was currently immune (i.e. had a symptomatic infection within the past 7 days or currently infected). A value of one denoted symptomatic infection for each day *j*, while asymptomatic infection, immunity, or non-infection was denoted with a value of zero for each day *j*. To calculate the estimated prevalence for a given day, the sum of column *j* was dived by the simulated population total (*n_i_*). Each source type and pathogen was modeled as a separate matrix.

|  |  | **Day *j*** | | |
| --- | --- | --- | --- | --- |
|  |  | ***j*** | … | ***j + n_d_*** |
| **Individual *i*** | ***i*** | … | … | … |
|  | … | … | … | … |
|  | ***i+n_i_*** | … | … | … |

**Fig E:** Schematic of matrix approach to simulate the longitudinal prevalence of symptomatic cryptosporidiosis and giardiasis, where each individual *i* in population *n_i_* infection status is modeled on day *j* for *n_d_* days.

**Fig F:** Risk profile plots of daily risk of *Cryptosporidium* (Cry.) and *Giardia* (Gia.) infection in deep and shallow tubewells for years 2012 and 2013 for under different scenarios used to assign the fraction of parasites infectious-to-humans (HUM, MST, ENV). Mean risk per day is printed for each scenario.

**Table F:** Kolmogorov-Smirnov two-sample statistic *P-values* between risk profiles for each parasite *Cryptosporidium* (Cry.) and *Giardia* (Gia.), tubewell type (deep = DTW and shallow = STW), and genotype scenario (HUM, ENV, MST) for monsoon season 2012, with *P-values* < 0.05 indicated with in bold.

| **Yr.**  **2012** | Cry.  DTW  HUM | Cry.  DTW  ENV | Cry.  DTW  MST | Gia.  DTW  HUM | Gia.  DTW  ENV | Gia.  DTW  MST | Cry.  STW  HUM | Cry.  STW  ENV | Cry.  STW  MST | Gia.  STW  HUM | Gia.  STW  ENV | Gia.  STW  MST |
| --- | --- | --- | --- | --- | --- | --- | --- | --- | --- | --- | --- | --- |
| Cry.  DTW  HUM | ------ | 0.054 | 0.906 | 1.000 | **<0.001** | 0.581 | 0.367 | **<0.001** | **0.002** | 0.699 | **<0.001** | **<0.001** |
| Cry.  DTW  ENV | 0.054 | ------ | 0.468 | 0.078 | 0.155 | 0.813 | 0.906 | 0.468 | 0.906 | 0.281 | **0.004** | 0.281 |
| Cry.  DTW  MST | 0.906 | 0.468 | ------ | 0.967 | **0.002** | 1.000 | 0.967 | **0.010** | 0.054 | 0.906 | **<0.001** | **0.004** |
| Gia.  DTW  HUM | 1.000 | 0.078 | 0.967 | ------ | **<0.001** | 0.699 | 0.468 | **<0.001** | **0.004** | 0.813 | **<0.001** | **<0.001** |
| Gia.  DTW  ENV | **<0.001** | 0.155 | **0.002** | **<0.001** | ------ | **0.010** | **0.010** | 0.994 | 0.813 | **0.001** | 0.699 | 0.967 |
| Gia.  DTW  MST | 0.581 | 0.813 | 1.000 | 0.699 | **0.010** | ------ | 0.994 | **0.037** | 0.155 | 0.967 | **<0.001** | **0.016** |
| Cry.  STW  HUM | 0.367 | 0.906 | 0.967 | 0.468 | **0.010** | 0.994 | ------ | **0.037** | 0.211 | 0.994 | **<0.001** | **0.024** |
| Cry.  STW  ENV | **<0.001** | 0.468 | **0.010** | **<0.001** | 0.994 | **0.037** | **0.037** | ------ | 0.994 | **0.004** | 0.367 | 1.000 |
| Cry.  STW  MST | **0.002** | 0.906 | 0.054 | **0.004** | 0.813 | 0.155 | 0.211 | 0.994 | ------ | **0.037** | 0.054 | 0.967 |
| Gia.  STW  HUM | 0.699 | 0.281 | 0.906 | 0.813 | **0.001** | 0.967 | 0.994 | **0.004** | **0.037** | ------ | **<0.001** | **0.002** |
| Gia.  STW  ENV | **<0.001** | **0.004** | **<0.001** | **<0.001** | 0.699 | **<0.001** | **<0.001** | 0.367 | 0.054 | **<0.001** | ------ | 0.367 |
| Gia.  STW  MST | **<0.001** | 0.281 | **0.004** | **<0.001** | 0.967 | **0.016** | **0.024** | 1.000 | 0.967 | **0.002** | 0.367 | ------ |

**Table G:** Kolmogorov-Smirnov two-sample statistic *P-values* between risk profiles for each parasite *Cryptosporidium* (Cry.) and *Giardia* (Gia.), tubewell type (deep = DTW and shallow = STW), and genotype scenario (HUM, ENV, MST) for monsoon season 2013, with *P-values* < 0.05 indicated with in bold.

| **Yr.**  **2013** | Cry.  DTW  HUM | Cry.  DTW  ENV | Cry.  DTW  MST | Gia.  DTW  HUM | Gia.  DTW  ENV | Gia.  DTW  MST | Cry.  STW  HUM | Cry.  STW  ENV | Cry.  STW  MST | Gia.  STW  HUM | Gia.  STW  ENV | Gia.  STW  MST |
| --- | --- | --- | --- | --- | --- | --- | --- | --- | --- | --- | --- | --- |
| Cry.  DTW  HUM | ------ | **0.004** | 0.054 | 0.211 | **<0.001** | **<0.001** | 0.906 | **<0.001** | 0.054 | 0.211 | **<0.001** | **<0.001** |
| Cry.  DTW  ENV | **0.004** | ------ | 0.967 | **0.024** | **<0.001** | **0.037** | **0.016** | 0.813 | 0.813 | 0.111 | **<0.001** | 0.468 |
| Cry.  DTW  MST | 0.054 | 0.967 | ------ | 0.211 | **<0.001** | **0.002** | 0.111 | 0.468 | 0.813 | 0.581 | **<0.001** | 0.054 |
| Gia.  DTW  HUM | 0.211 | 0.024 | 0.211 | ------ | **<0.001** | **<0.001** | 0.367 | **0.002** | 0.111 | 0.906 | **<0.001** | **<0.001** |
| Gia.  DTW  ENV | **<0.001** | **<0.001** | **<0.001** | **<0.001** | ------ | **0.002** | **<0.001** | **<0.001** | **<0.001** | **<0.001** | 0.813 | **<0.001** |
| Gia.  DTW  MST | **<0.001** | **0.037** | **0.002** | **<0.001** | **0.002** | ------ | **<0.001** | 0.155 | **0.002** | **<0.001** | **0.001** | 0.813 |
| Cry.  STW  HUM | 0.906 | **0.016** | 0.111 | 0.367 | **<0.001** | **<0.001** | ------ | **0.004** | 0.211 | 0.281 | **<0.001** | **<0.001** |
| Cry.  STW  ENV | **<0.001** | 0.813 | 0.468 | **0.002** | **<0.001** | 0.155 | **0.004** | ------ | 0.581 | **0.016** | **<0.001** | 0.813 |
| Cry.  STW  MST | 0.054 | 0.813 | 0.813 | 0.111 | **<0.001** | **0.002** | 0.211 | 0.581 | ------ | 0.468 | **<0.001** | 0.054 |
| Gia.  STW  HUM | 0.211 | 0.111 | 0.581 | 0.906 | **<0.001** | **<0.001** | 0.281 | 0.016 | 0.468 | ------ | **<0.001** | **0.001** |
| Gia.  STW  ENV | **<0.001** | **<0.001** | **<0.001** | **<0.001** | 0.813 | **0.001** | **<0.001** | **<0.001** | **<0.001** | **<0.001** | ------ | **<0.001** |
| Gia.  STW  MST | **<0.001** | 0.468 | 0.054 | **<0.001** | **<0.001** | 0.813 | **<0.001** | 0.813 | 0.054 | **0.001** | **<0.001** | ------ |

**Table H:** Simulated mean seasonal daily child diarrhea prevalence (%) (median and (25^th^ and 75^th^ percentiles)) under the HUM, MST, and ENV scenarios for each water source, year, and pathogen.

| Source | Year | Pathogen | HUM Prevalence median (25^th^-75^th^) | MST Prevalence median (25^th^-75^th^) | ENV Prevalence median (25^th^-75^th^) |
| --- | --- | --- | --- | --- | --- |
| Deep tubewell | 2012 | *Cryptosporidium* | 4.51 (4.08, 5.11) | 2.99 (2.61, 3.33) | 1.58 (1.30, 1.79) |
|  |  | *Giardia* | 4.95 (4.57, 5.33) | 2.45 (2.17, 2.72) | 0.33 (0.27, 0.49) |
| Shallow tubewell |  | *Cryptosporidium* | 1.86 (1.60, 2.16) | 0.65 (0.54, 0.80) | 0.50 (0.40, 0.65) |
|  |  | *Giardia* | 1.91 (1.71, 2.11) | 0.25 (0.15, 0.30) | 0.05 (0.00, 0.10) |
| Deep tubewell | 2013 | *Cryptosporidium* | 0.87 (0.71, 1.03) | 0.27 (0.22, 0.38) | 0.22 (0.11, 0.27) |
|  |  | *Giardia* | 0.16 (0.11, 0.22) | 0.00 (0.00, 0.05) | 0.00 (0.00, 0.00) |
| Shallow tubewell |  | *Cryptosporidium* | 0.75 (0.65, 0.90) | 0.30 (0.25, 0.40) | 0.20 (0.15, 0.25) |
|  |  | *Giardia* | 0.20 (0.10, 0.25) | 0.05 (0.00, 0.05) | 0.00 (0.00, 0.00) |

**Table I:** Simulated 7-day recall child diarrhea period prevalence (%) (median and (25^th^ and 75^th^ percentiles)) under the HUM, MST, and ENV scenarios for each water source, year, and pathogen.

| Source | Year | Pathogen | HUM Prevalence mean (25^th^-75^th^) | MST Prevalence mean (25^th^-75^th^) | ENV Prevalence mean (25^th^-75^th^) |
| --- | --- | --- | --- | --- | --- |
| Deep tubewell | 2012 | *Cryptosporidium* | 5.65 (5.27, 6.09) | 3.70 (3.37, 4.02) | 1.90 (1.68, 2.12) |
|  |  | *Giardia* | 6.03 (5.71, 6.41) | 3.04 (2.77, 3.32) | 0.43 (0.33, 0.54) |
| Shallow tubewell |  | *Cryptosporidium* | 2.31 (2.11, 2.61) | 0.85 (0.70, 1.00) | 0.65 (0.55, 0.80) |
|  |  | *Giardia* | 2.36 (2.16, 2.61) | 0.30 (0.20, 0.40) | 0.10 (0.05, 0.15) |
| Deep tubewell | 2013 | *Cryptosporidium* | 1.03 (0.87, 1.25) | 0.33 (0.27, 0.43) | 0.27 (0.16 0.33) |
|  |  | *Giardia* | 0.22 (0.16, 0.27) | 0.00 (0.00, 0.05) | 0.00 (0.00, 0.00) |
| Shallow tubewell |  | *Cryptosporidium* | 0.95 (0.84, 1.15) | 0.40 (0.30, 0.50) | 0.25 (0.15, 0.35) |
|  |  | *Giardia* | 0.25 (0.15, 0.30) | 0.05 (0.00, 0.05) | 0.00 (0.00, 0.00) |

**References**

1. Haas CN, Rose JB, Gerba CP. Quantitative microbial risk assessment: John Wiley & Sons; 1999.

2. Teunis PFM, Chappell CL, Okhuysen PC. *Cryptosporidium* dose response studies: variation between isolates. Risk Anal. 2002;22(1):175-85.

3. Teunis PFM, Havelaar AH. *Cryptosporidium* in drinking water: evaluation of the ILSI quantitative risk assessment framework. 1999.

4. Rose JB, Haas CN, Regli S. Risk assessment and control of waterborne giardiasis. Am J Public Health. 1991;81(6):709-13.

5. Givens GH, Hoeting JA. Computational statistics: John Wiley & Sons; 2012.

6. R Development Core Team. R: A language and environment for statistical computing. Vienna, Austria: R Foundation for Statistical Computing; 2017.

7. Brooks SP, Gelman A. General methods for monitoring convergence of iterative simulations. J Comput Graph Stat. 1998;7(4):434-55.

8. Petterson SR, Signor RS, Ashbolt NJ. Incorporating method recovery uncertainties in stochastic estimates of raw water protozoan concentrations for QMRA. J Water Health. 2007;5(1):51.

9. WHO G. Guidelines for drinking-water quality. World Health Organization. 2011;216:303-4.

10. Daniels ME, Shrivastava A, Smith WA, Sahu P, Odagiri M, Misra PR, et al. *Cryptosporidium* and *Giardia* in Humans, Domestic Animals, and Village Water Sources in Rural India. Am J Trop Med Hyg. 2015:596-600.

11. Kang G, Mathew MS, Rajan DP, Daniel JD, Mathan MM, Mathan VI, et al. Prevalence of intestinal parasites in rural Southern Indians. Trop Med Int Health. 1998;*3*(1):70-5.

12. Government of Odisha. Orissa Review (Census Special) 2010 [2016]. Available from: <http://odisha.gov.in/e-magazine/Orissareview/2010/December/engpdf/172-175.pdf>.

13. Pyramid Population. India 2016 2016 [cited 2016]. Available from: <https://populationpyramid.net/india/2013/>.

14. Rose C, Parker A, Jefferson B, Cartmell E. The characterization of feces and urine: A review of the literature to inform advanced treatment technology. Crit Rev Environ Sci Technol. 2015;45(17):1827-79.

15. Odagiri M, Schriewer A, Daniels ME, Wuertz S, Smith WA, Clasen T, et al. Human fecal and pathogen exposure pathways in rural Indian villages and the effect of increased latrine coverage. Water Res. 2016.

16. Teunis PFM, Medema GJ, Kruidenier L, Havelaar AH. Assessment of the risk of infection by *Cryptosporidium* or *Giardia* in drinking water from a surface water source. Water Res. 1997;31(6):1333-46.

17. LeChevallier MW, Norton WD, Lee RG. Occurrence of *Giardia* and *Cryptosporidium* spp. in surface water supplies. Appl Environ Microbiol. 1991;57(9):2610-6.

18. Environmental Protection Agency U.S. Child-specific exposure factors handbook. In: USEPA, editor.: Office of Research and Development, US Environmental Protection Agency Washington, DC; 2011.

19. Hossain MA, Rahman MM, Murrill M, Das B, Roy B, Dey S, et al. Water consumption patterns and factors contributing to water consumption in arsenic affected population of rural West Bengal, India. Sci Total Environ. 2013;463:1217-24.

20. Sarkar R, Ajjampur SSR, Prabakaran AD, Geetha JC, Sowmyanarayanan TV, Kane A, et al. Cryptosporidiosis among children in an endemic semiurban community in southern India: does a protected drinking water source decrease infection? Clin Infect Dis. 2013;57(3):398-406.

21. Cordón GP, Soldan OCP, Vásquez FV, Soto JRV, Bordes LS, Moreno MS, et al. Prevalence of enteroparasites and genotyping of *Giardia* *lamblia* in Peruvian children. Parasitol Res. 2008;103(2):459-65.

22. Platts-Mills JA, Babji S, Bodhidatta L, Gratz J, Haque R, Havt A, et al. Pathogen-specific burdens of community diarrhoea in developing countries: a multisite birth cohort study (MAL-ED). *Lancet Glob Health*. 2015;3(9):e564-e75.

23. Liu J, Platts-Mills JA, Juma J, Kabir F, Nkeze J, Okoi C, et al. Use of quantitative molecular diagnostic methods to identify causes of diarrhoea in children: a reanalysis of the GEMS case-control study. The Lancet. 2016;388(10051):1291-301.

24. Insulander M, Lebbad M, Stenström TA, Svenungsson B. An outbreak of cryptosporidiosis associated with exposure to swimming pool water. Scand J Infect Dis Suppl. 2005;37(5):354-60.

25. Cicirello HG, Kehl KS, Addiss DG, Chusid MJ, Glass RI, Davis JP, et al. Cryptosporidiosis in children during a massive waterborne outbreak in Milwaukee, Wisconsin: clinical, laboratory and epidemiologic findings. Epidemiol Infect. 1997;119(01):53-60.

26. Yamamoto N, Urabe Ki, Takaoka M, Nakazawa K, Gotoh A, Haga M, et al. Outbreak of cryptosporidiosis after contamination of the public water supply in Saitama Prefecture, Japan, in 1996. *Kansenshogaku Zasshi*. 2000;74(6):518-26.

27. Cristino JAGM, Isabel M, Carvalho P, Salgado MJ. An outbreak of cryptosporidiosis in a hospital day-care centre. Epidemiol Infect. 1988;101(02):355-9.

28. Enger KS, Nelson KL, Clasen T, Rose JB, Eisenberg JNS. Linking quantitative microbial risk assessment and epidemiological data: informing safe drinking water trials in developing countries. Environ Sci Technol. 2012;46(9):5160-7.
